# Routine Health Information Systems in the European Context: A Systematic Review of Systematic Reviews

**DOI:** 10.3390/ijerph18094622

**Published:** 2021-04-27

**Authors:** Francesc Saigí-Rubió, José Juan Pereyra-Rodríguez, Joan Torrent-Sellens, Hans Eguia, Natasha Azzopardi-Muscat, David Novillo-Ortiz

**Affiliations:** 1 Faculty of Health Sciences, Universitat Oberta de Catalunya (UOC), 08018 Barcelona, Spain; fsaigi@uoc.edu (F.S.-R.); heguia@uoc.edu (H.E.); 2 Interdisciplinary Research Group on ICTs, 08035 Barcelona, Spain; jtorrent@uoc.edu; 3 Department of Dermatology, University Hospital Virgen del Rocío, 41013 Sevilla, Spain; pe3reyra@gmail.com; 4 Faculty of Economics and Business, Universitat Oberta de Catalunya (UOC), 08035 Barcelona, Spain; 5 SEMERGEN New Technologies Working Group, 28009 Madrid, Spain; 6 Division of Country Health Policies and Systems, Regional Office for Europe, World Health Organization, 2100 Copenhagen, Denmark; muscatn@who.int

**Keywords:** routine health information system, health management information system, health system performance

## Abstract

(1) Background: The aim of this study is to provide a better understanding of the requirements to improve routine health information systems (RHISs) for the management of health systems, including the identification of best practices, opportunities, and challenges in the 53 countries and territories of the WHO European region. (2) Methods: We conducted an overview of systematics reviews and searched the literature in the databases MEDLINE/PubMed, Cochrane, EMBASE, and Web of Science electronic databases. After a meticulous screening, we identified 20 that met the inclusion criteria, and RHIS evaluation results were presented according to the Performance of Routine Information System Management (PRISM) framework. (3) Results: The reviews were published between 2007 and 2020, focusing on the use of different systems or technologies and aimed to analyze interventions on professionals, centers, or patients’ outcomes. All reviews examined showed variability in results in accordance with the variability of interventions and target populations. We have found different areas for improvement for RHISs according to the three determinants of the PRISM framework that influence the configuration of RHISs: technical, organizational, or behavioral elements. (4) Conclusions: RHIS interventions in the European region are promising. However, new global and international strategies and the development of tools and mechanisms should be promoted to highly integrate platforms among European countries.

## 1. Introduction

High-quality data supporting health management decisions are key to effective governance, leadership, and management [1,2,3,4,5,6]. Informational support for all levels of health management enables planning, policymaking, operational management, and continuous quality improvement [2]. A health information system (HIS) is a set of components (technical, organizational, behavioral) and procedures “organized to generate information that allows improving health management decisions at all levels of the health system” [7]. When a HIS produces high-quality, timely, and reliable data, it enables health program managers to monitor, evaluate, and improve health system performance and make evidence-based decisions. This information can then aid decision making, including the prioritization of funding and the allocation of other resources, and to assess which information or sources of in-formation are missing, uncertain, or of low quality [8]. These data can be used to system-atically explore new ideas, while formulating basic strategies to support them (WHO Eu-ropean Health Information Initiative (EHII)) [8].

Healthcare providers routinely collect data on health services, statuses, and re-sources. In turn, public health advisors, hospital and healthcare managers, and ongoing surveys of health facilities also provide information. The data provide a snapshot of the state of health, health services, and health resources. The sources of these data are generally records of services rendered, individual medical records, and records of health resources. They provide information about the health of the patients and the type of treatments and tests they receive. Other information may be collected by managers on human resources, finances, drugs, and supply systems.

Routine medical information may originate from a variety of data sources that include information related to the provision of clinical services (e.g., clinical records, laboratory, and other diagnostic systems service records) and administrative record systems of routine (e.g., staff timesheets), which can be collected during regular periods (daily, monthly, quarterly, annually). A routine health information system (RHIS, also called a health facility and community information system) is any system of data collection, distribution, and use that provides information at regular intervals that is produced through routine mechanisms to address predictable health information needs [9]. Routine data on health service delivery, utilization, and clinical outcomes are reported more frequently, but an RHIS also includes routine data sets related to other health system functions (human resources management, finance, drug and equipment supply chains, and governance and management) [1].

RHISs generate data at regular intervals (one year or less) that have been collected from public and private health facilities and institutions, and community-level healthcare posts and clinics. An RHIS effectively and efficiently supports management decision making if it produces good quality data with timely, relevant, accurate, complete, and accessible information. If this is the case, optimal impact can be achieved in health outcomes and the functioning of health systems. The data produced by RHISs allow evidence-based decisions to be made for the governance and management of health systems and services for planning, monitoring and evaluation, and quality improvement.

An effective RHIS has two main objectives: first, to produce high-quality, routine health information; and second, the effective use of routine health information for decision making [7,10,11]. The ultimate objective of an RHIS is not information for its own sake but to “improve health services management through optimal informational support” [7]. A robust RHIS can be achieved by improving data production (data quality and accessibility) or data use (the capacity and processes for effective, data-informed decision making).

Given the centrality of routine information to management decision making and the challenge of making decisions when these systems are not optimal, we need to know what works in which settings for RHISs to support health system management decision-making effectively [7,12,13,14,15]. Synthesized evidence from research studies that evaluated interventions to address this challenge can help offer solutions to improve RHISs, and in turn to strengthen health system management.

An RHIS mainly focuses on high-level information management (national, regional, and district levels) without obligatory feedback to lower levels such as physicians. This stratification is counterproductive because, in cases of emergencies, pandemics, or natural disasters, those health personnel are the first in contact with the population. Thus, RHIS at local, provincial or state, and national levels need to be strengthened, so they can provide relief personnel with up-to-date information for planning [16].

RHISs also can assist physicians in making evidence-based decisions to enhance the local health system’s performance. Positive health outcomes can be improved with the right implementation of an RHIS at both the hospital and primary care levels [17]. An optimally functioning RHIS could remove obstacles between individual care and public health information systems, ultimately improving individuals’ health statuses and strengthening the global health system with more effective and efficient management and planning.

The Performance of Routine Information System Management (PRISM) framework is an innovative approach to designing, strengthening, and evaluating RHIS performance by incorporating organizational, technical, and behavioral determinants of performance [12]. The PRISM framework identifies two main functions of an RHIS and three key domains that are influential in shaping RHIS. The two main functions of an RHIS are the production of quality data and the effective use of data for decision making. These three key domains also represent areas for improving RHIS:

Technical: Technical interventions to improve an RHIS are usually intended to improve the design and the technical aspects of the RHIS, such as the usefulness and functionality of registers and computer hardware and software.

Behavioral: Behavioral interventions aim to improve staff motivation and skills to collect, extract, and use data effectively.

Organizational: Organizational interventions are meant to strengthen organizational rules, values, and support practices aimed at building a culture of data use for decision making.

RHIS interventions can address any of the components described in the PRISM framework [12,13]. An example of using multiple data streams for disease surveillance is influenza surveillance [18].

In this systematic review, we recognize that reliable health information and data that are embedded in a fully functioning and high-quality HIS form the foundation for sound decision making in healthcare and are essential for health system policy development. The aim of this systematic review is to provide a better understanding of the requirements to improve RHIS for the management of health systems, including the identification of best practices, opportunities, and challenges in the 53 countries and territories of the WHO European region.

The article makes a new contribution, from a number of perspectives, to the literature on this topic. Firstly, a systematic review of mostly Europe-centered literature is performed, taking into consideration the multidimensional set of routine practices undertaken within the HIS context. This has involved the conceptualization and delimitation of RHIS within the HIS family. Moreover, a PRISM framework approach has been taken to the literature review. This framework is widely used in the literature on the topic of HIS technologies but is rarely used within the context of RHIS. In this regard, a set of drivers and, in particular, barriers have been identified. These barriers limit the use of RHIS and the generation of RHIS-based outputs and outcomes. Lastly, this review makes a unique contribution because it supplements the results identified in the literature in two ways. First, it analyzes the link between RHIS and new health management systems based on big data or machine-learning behavior prediction algorithms. Second, it reflects on how RHISs have helped in managing the COVID-19 pandemic.

## 2. Materials and Methods

### 2.1. Search Strategy and Inclusion Criteria

This study is a systematic review of reviews that assessed “data collection” and “health information system assessments” with a focus on routine health information systems (RHISs). The study was conducted in accordance with the AMSTAR 2 [19] checklists and Preferred Reporting Items for Systematic Reviews and Meta-Analyses (PRISMA) statement [20] to ensure the quality of the review and the methodological considerations when using existing systematic reviews. It has been conveniently registered in the PROSPERO database with the number CRD42020207267. The risk of bias was assessed, and disagreements regarding bias and the interpretation of results were resolved by consensus discussions.

A literature search was performed using MEDLINE (accessed through PubMed), Cochrane (Cochrane Database of Systematic Reviews, Cochrane Central Register of Controlled Trials, Cochrane Methodology Register, Database of Abstracts of Reviews of Effects, National Health Service Economic Evaluation Database), EMBASE, and Web of Science electronic databases in August 2020, using the following set of keywords:

Routine Health Information Systems:

“Health informatics” [TIAB] OR “health information system *” [TIAB] OR “hospital information system *” [TIAB] OR “management information system *” [TIAB] OR “ambulatory care information system *” [TIAB] OR “clinical laboratory information system *” [TIAB] OR “clinical pharmacy information system *” [TIAB] OR “radiology information system *” [TIAB] OR “medical order entry system *” [TIAB] OR “health information management” [TIAB] OR “decision support system *” [TIAB] OR “health information exchange” [TIAB] OR “interoperability” [TIAB] OR “information system *” [TIAB] OR “medical informatic *” [TIAB] OR “dental informatic *” [TIAB] OR “health information” [TIAB] OR “nursing informatic *” [TIAB] OR “public health informatic *” [TIAB] OR “medical record *” [TIAB] OR “electronic health record *” [TIAB] OR “personal health record *” [TIAB] OR “individual health record *” [TIAB] OR “RHIS” [TIAB] OR “routine health information system *” [TIAB] OR “eHealth” [TIAB] OR “e-Health” [TIAB].

WHO European region (53 countries and territories):

“Albania” [TIAB] OR “Andorra” [TIAB] OR “Armenia” [TIAB] OR “Austria” [TIAB] OR “Azerbaijan” [TIAB] OR “Belarus” [TIAB] OR “Belgium” [TIAB] OR “Bosnia and Herzegovina” [TIAB] OR “Bulgaria” [TIAB] OR “Croatia” [TIAB] OR “Cyprus” [TIAB] OR “Czechia” [TIAB] OR “Denmark” [TIAB] OR “Estonia” [TIAB] OR “Finland” [TIAB] OR “France” [TIAB] OR “Georgia” [TIAB] OR “Germany” [TIAB] OR “Greece” [TIAB] OR “Hungary” [TIAB] OR “Iceland” [TIAB] OR “Ireland” [TIAB] OR “Israel” [TIAB] OR “italy” [TIAB] OR “Kazakhstan” [TIAB] OR “Kyrgyzstan” [TIAB] OR “Latvia” [TIAB] OR “Lithuania” [TIAB] OR “Luxembourg” [TIAB] OR “Malta” [TIAB] OR “Monaco” [TIAB] OR “Montenegro” [TIAB] OR “Netherlands” [TIAB] OR “North Macedonia” [TIAB] OR “Norway” [TIAB] OR “Poland” [TIAB] OR “Portugal” [TIAB] OR “Moldova” [TIAB] OR “Romania” [TIAB] OR “Russia” [TIAB] OR “San Marino” [TIAB] OR “Serbia” [TIAB] OR “Slovakia” [TIAB] OR “Slovenia” [TIAB] OR “Spain” [TIAB] OR “Sweden” [TIAB] OR “Switzerland” [TIAB] OR “Tajikistan” [TIAB] OR “Turkey” [TIAB] OR “Turkmenistan” [TIAB] OR “Ukraine” [TIAB] OR “United Kingdom” [TIAB] OR “Uzbekistan”.

The search was restricted to systematic reviews, by publication date (from 1 January 2000 up to 15 August 2020), and by publication language (English and Spanish).

### 2.2. Study Selection

The systematic review includes data from reviews that covered any practice targeting any component or dimension of an RHIS, with at least one component related to health services performance or management in at least one WHO European country or territory. Exclusion criteria were (1) studies written in languages other than English, and those for which the full text was not available online; and (2) conference abstracts.

Initial screening was based on titles and abstracts by three researchers (J.J.P.-R, J.T.-S., and F.S.-R.). Disagreement on bias assessment and the interpretation of results was resolved by two investigators (D.N.-O. and H.E.). Abstracts lacking information were retrieved for full-text evaluation. Subsequently, the same investigators independently evaluated full-text articles and determined eligibility. Disagreement on bias assessment and the interpretation of results was resolved by consensus discussions. Authorship, journal, and years were not blinded.

### 2.3. Data Extraction and Quality Assessment

Three investigators conducted data extraction following standardized criteria, and results were reviewed by two senior researchers. The following data were extracted: journal, publication year, databases searched, time period, setting, system or technology, data type and collection, intervention type, number of studies, total number and countries of patients, study design, whether a review of systematic reviews or meta-analysis or bibliometric analysis was performed, outcomes, lessons and barriers for implementation, main results, main limitations, implications: challenges and opportunities, and information systems evaluation (see Tables S1 and S2 in Supplementary Materials).

## 3. Results

**Figure 1:**
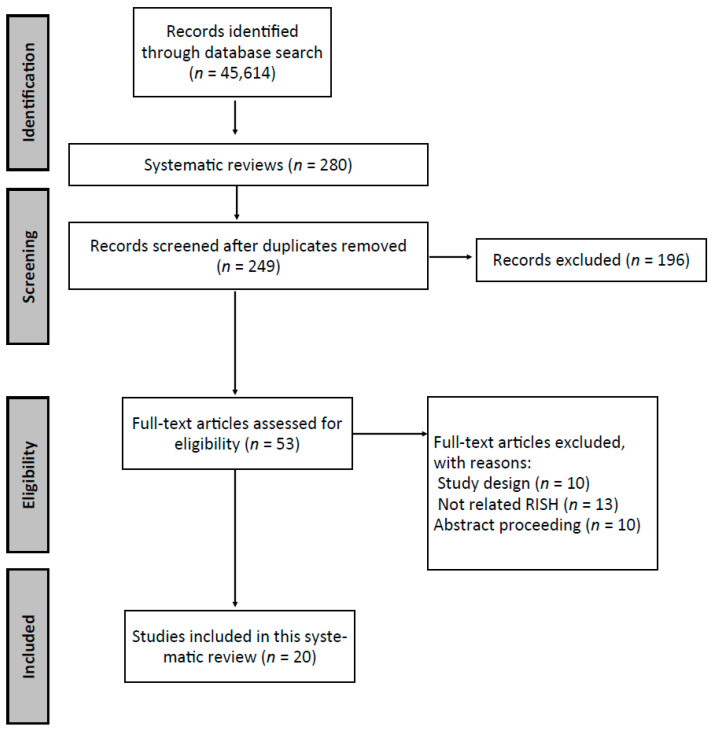
Flow of information through the different phases of the systematic review.

A flow chart of the literature search and study selection results is shown in Figure 1. The first database search resulted in 45,614 articles; the updated search resulted in 280 articles. After exclusion of duplicates, 249 articles were screened, and 196 were excluded. Full texts of 53 eligible articles were reviewed. Out of these, 33 were excluded for not meeting the criteria relating to study type, intervention, or outcome. The 20 remaining studies were included in this systematic review.

### 3.1. Descriptive Analysis of the Systematic Reviews

#### 3.1.1. General Characteristics of Reviewed Papers

The 20 systematic reviews included in our review were published between 2007 and 2020 in 12 unique journals. In these reviews, systematic literature searches were performed from 1974 to 2019, and all reviews were international (covering between 3 and 14 countries). The system or technology analyzed was varied, the most frequent being general ICT systems, medical health records, automated alert and reminder systems, and support systems for clinical decision making. The most frequently applied setting on which the technology focused and aligned were hospital care, primary care, and emergency services; two studies focused on aging, and one on AIDS and hypertension.

Almost all the studies included a multidatabase search, except for Anker et al., who only searched PsychInfo [21], and Marschollek, who searched PubMed [22]. The number of studies included in the systematic reviews ranged between 4 and 99, the majority between 20 and 40 studies. Only 2 of the 20 systematic reviews also included a meta-analysis [23,24] (see Table S1 in Supplementary Materials).

The studies included in the systematic reviews were diverse. Most included both randomized and nonrandomized clinical trials, including retrospective case series, case–controls, descriptive cohorts, and qualitative studies.

#### 3.1.2. Aims

Most of the reviews aimed to analyze the impact of interventions on the outcomes of the professional (readmission), centers (drug alerts, patient decisions), or of the patients (independent aging, healthy behaviors). Some studies analyzed factors that generally influenced practitioners in the use of patient data collection applications. One article explored the barriers and facilitators in the use of health information exchange systems.

#### 3.1.3. Intervention

The reviews included studies with interventions based on different technologies or systems. Most of the studies were based on EHR and contextual patient information in intensive and emergency care [25,26,27]; ambulatory or primary care [25,28,29]; healthcare settings, including hospitals [30]; patient results, performance, and safety [31]; and prescription alerts via EHR [23]. Other systems evaluated were health smart homes (HSHs) and home-based consumer health (HCH) for the activity of elderly people [32], and clinical decision support for the management of AIDS [33]. Other reviews included combinations of several systems, such as CDSS, computerized provider order entry (CPOE), and electronic prescribing [24,34]. Lastly, some reviews analyzed generic RHISs [35,36] (see Table S1 in Supplementary Materials).

### 3.2. Outcomes

The reviews naturally showed variability in results in accordance with the variability of interventions and target populations. Studies that focused on evaluating an intervention generally show weak evidence in favor of its use. This occurs, for example, in the Arditi [29] reminder study, in which they concluded that reminders to professionals can probably improve the quality of care in various contexts and under various conditions. Even studies about some interventions, such as technologies for independent aging [32], did not find strong evidence to support the technology.

Some reviews analyzed the use of different clinical information systems in different settings [24,25,27,30,31,33,35,36]. These will likely provide the most encouraging results. Several studies conclude that using an RHIS makes it possible to improve efficiency both in management (reduction of missed appointments, waiting times, etc.) [33] and for clinicians (better communication with patients and colleagues, patient information in real time), which allows better coordination, decision making, and health outcomes [30,36].

Some studies value RHISs as administrative, public health, or epidemiology tools and also consider them useful assets for various medical specialties such as emergency and critical care in hospital medicine or primary care (GP clinics) [23,25,26,28,29,31]. Studies also analyzed communication systems between patients and healthcare workers, particularly nurses. From the results obtained, ICTs showed to improve the nurse–patient relationship and increase empowerment, knowledge, well-being, and even the state of health [37].

A study analyzed the employment of health-smart homes (HSHs) and home-based consumer health (HCH) technologies to support aging at home [32], but due to the design and quality of the studies—sample sizes, etc.—there was insufficient evidence to support the role of these systems in improving independent living in the homes of the elderly. The systems used in monitoring older adults do not adequately collect or are not designed for the purpose of being assessed by an RHIS. The collected data mostly reflects the patient’s current status and is then discarded [22].

Baysari et al. used information technology as decision-making support systems integrated into EMR decisions for prescribing antibiotics [24]. These systems can help improve the use of antibiotics in the hospital environment. However, there is mixed evidence of the impact on final health outcomes such as mortality or length of stay. Great variability was also found in the designs of the studies; therefore, more evidence is needed to conclude that these systems can help organizations improve their prescribing. Bayoumi et al. also evaluated computerized alerts to improve prescribing [23]. Analyzed results showed a reduction of adverse events and hospitalization; clinical outcomes such as reduction in hypoglycemia and optimization in the maintenance of INR in therapeutic range for anticoagulants; and finally, changes in prescription behavior, which had the most immediate impact and evidence. This means that an RHIS can also affect medical audits by validating probable errors in medication [38], laboratory results [33], undeclared medication side effects [23], etc.

The use of different software applications for data collection [28] and the hesitancy to share health data with competitors [25]—especially in countries where health systems are private—are major drawbacks in global data generation. Hence, it should be recognized that RHISs follow strong privacy and safety protections for ethical use and collection of useful information. Unfortunately, some information sources such as EHR failed to present adequate or correctly used data [25]; in some cases, doctors inputted data poorly because of low computer literacy [33]. With these problems solved, RHISs would also be useful for improving access to information by making it more visible and contextualized [27]. Educating health personnel on the correct management of EHRs could alleviate this problem [28,37].

Other initiatives, such as the development of strategic frameworks, clinical leadership that values technology skills [31,36], financial resources for training [35], and the development of strategies to overcome resistance to change in health personnel [24,34] could improve the RHIS’s ability to gather better information.

Another group of interventions analyzed focused on ICTs in general, as well as the use of the internet and social media [21,22,37].

Effective RHIS function requires the interaction between physicians, technical personnel, technology, the clinical environment, and the social system to work [27], along with the correct data input, adequate policies, and leadership from key players in the system. Table S3 in Supplementary Materials shows an overview of the attributes of the dimensions of success measured in the 20 systematic reviews.

### 3.3. Areas for Improvement for RHISs According to the PRISM Framework

To evaluate RHIS, we used the PRISM framework. This conceptual framework hypothesizes that technical, behavioral, and organizational determinants (inputs) influence data collection, transmission, processing, and presentation (Table 1). These, in turn, influence data quality and use (outputs), which include technical, organizational, and behavioral aspects related to the effective use of information for decision making (Table 2), health system performance (outcomes), and ultimately, health outcomes that represent a health impact (Table 3) [13]. According to the three determinants of the PRISM framework that influence the configuration of RHIS (technical, organizational, or behavioral elements) we have found the following areas for improvement for RHISs.

Regarding the inputs, the literature review shows that there is a set of actions that could foster more efficient and effective use of RHISs. Firstly, the use of contextual frameworks or theoretical models would enable an analysis of RHIS use-related behavior to be performed. One of the problems identified in the literature is the lack of theoretical references in the explanation of RHIS acceptance by healthcare professionals [25,36]. Linked to this first element, the review has also highlighted the need for a much better connection between RHIS use and people’s skills and organizations’ abilities [24,25,26,27,37,39]. Relationships of complementarity between RHIS, healthcare professionals’ competencies and skills, and less bureaucratic organizational forms that are better adapted to evidence-based decision making [21,31,32,34] are also especially important when it comes to fostering RHIS use. Additionally, third, from the input perspective, the literature also highlights the need to overcome the technical and technological limitations that undermine the effective use of RHISs [25,28,30]. Among such limitations are problems associated with connectivity, bandwidth, usability, and interoperability between systems [35].

Regarding RHIS use-related outputs, the review also points to a set of elements that could facilitate more effective uses and returns. Firstly, a whole set of elements linked to data management has been emphasized. The management of privacy, security, and confidentiality of RHIS health data input and output is of vital importance [25]. Within this context, the importance of developing confidentiality protocols that are compatible with the use of data for evidence-based decision making has been noted [31]. In addition, issues linked to the security and adaptability (e.g., to generational preferences [22,32,35]) of RHIS input data collection and storage devices have also been emphasized [37,40].

Regarding RHIS outcomes, the literature review also offers some relevant conclusions. First, it is important to note that, despite the importance of using RHIS to support evidence-based decision making in health systems, the available evidence on its outcomes is very limited to analyses of effectiveness in specific areas [23,33,38]. There is little evidence of findings on the effects of RHIS use for health systems as a whole [21,23,24,29,30]. Second, and taking into account the reluctance to use RHISs and the limitations of the information obtained from them, the review also highlights the need to incorporate the needs of professionals who use RHISs [27,34,36]. Once again, this leads us to the question of relationships of complementarity with people and organizations [27,39]. To ensure that RHISs have efficient and effective outcomes, it is vital to consider both healthcare professionals’ digital competencies and information management skills, as well as a flattening of organizational hierarchies and “top-down” mechanisms [27,34,35].

## 4. Discussion

RHISs are an evolution of HISs. Much broader in scope, they are complex, nested systems for health data collection and management. The novelty of RHISs rests on two main elements: the regularity of data captured and the effective use of these data for decision making. With these two novel elements, RHISs facilitate data production and enable isolated data-driven decisions to be made. The aim is to provide support for integral decision making in healthcare through information systems containing regular, optimal data.

To evaluate RHISs, we used the PRISM framework. This conceptual framework is useful for evaluating the effectiveness of an RHIS by defining and relating its inputs, outputs, and outcomes. The PRISM framework draws a flow diagram in which:

1. Based on an intervention in the HIS, a set of technological, organizational, and behavioral drivers and barriers arise;
2. The interaction between the intervention and the drivers and barriers generates RHIS inputs, i.e., the data that will be used. To achieve this, the data’s needs, production, availability, and use requirements must be precisely defined;
3. Once the data have been generated, they are transformed into RHIS outputs, to the extent that they can generate high-quality health information, and then that health information is used effectively for decision making;
4. Once health information has been generated and used effectively, the RHIS is ready to generate outcomes, i.e., the results of its implementation. In general, these results refer to the effectiveness of either the information system itself or the health system in general. The ultimate intention is to improve citizens’ health statuses.

Through PRISM and a systematic literature review of 20 scientific articles that reviewed the literature on the various practical dimensions of HISs, we reached the following main conclusions:

### 4.1. Inputs

We have found four key aspects that need to be improved:

First, RHISs need to incorporate new underlying frameworks to predict behaviors for adoption and use. In this context, and with regard to modeling, it would be useful to have updates of the unified theory of acceptance and use of technology (UTAUT) [41], the theory of planned behavior (TPB) [42,43], the theory of diffusion of innovations (DOI) [44], and the theory of organization and environment (TOE) [45]. These frameworks have all been used in literature that investigates the motivations for the use of technology in various contexts, including healthcare [46,47,48,49,50,51].

Second, the usability and interoperability between RHISs and their ability to connect with each other need to be improved considerably. In addition, the choice of the HIS provider is key for their subsequent development. In this respect, health organizations must gain a better understanding of the information systems market in general and the HIS market in particular.

Third, the changing nature of information systems and technology use suggests improvement in some aspects of organizations. One example is training professionals in digital skills, in information systems in general and HIS in particular, such as training physicians to input data more accurately into the EHR. Medical professionals must prioritize developing skills in transformational leadership and management of healthcare organizations, crucial to overcoming probable resistance present in some healthcare professionals. Additionally, collaborative networks must be created between technical and healthcare professionals in the context of HISs. Furthermore, organizational culture must be developed among healthcare personnel to make evidence-based decisions in healthcare organizations, and, in particular, in the evaluation of healthcare policies. Additionally, organizations must establish investment-financing mechanisms because of the economic effort involved in developing and maintaining RHISs, including public–private partnerships and learning from the experiences of other sectors. Furthermore, it is advisable to promote connections between medical science systems and information systems and technologies. The connection between medical research and the medical device market is well developed, but the same cannot be said for the connection between medical research and HIS development. Connecting the medical research, technology, and management sectors is crucial for the efficient and useful development and implementation of RHISs. In this sense, the creation of a specific training agreement would be useful. Finally, operational groups and tasks of the data scientists office (DSO) must be incorporated into health organizations and public health policy evaluation teams.

Fourth, RHISs also have information and communication infrastructure requirements. However advanced the HIS might be, it cannot be effective within contexts with connectivity and bandwidth problems. In this regard, 5G technology offers possibilities.

### 4.2. Outputs

Issues of privacy, confidentiality, and security of the data generated and used in RHISs are of vital importance. RHISs should reinforce the protocols ensuring that any data obtained are used confidentially and securely, without limiting their potential to be used to improve decision making in healthcare.

The emergence of big data and data-driven management is a great opportunity for RHISs. Unlike earlier methods, big data allows initially unstructured mass data to be collected and processed. For example, through social listening methodologies that can be matched to clinical and behavioral data, healthcare management can have access to broader, more accurate, and robust information about any dimension of health. This is especially important in the acute management of situations such as the coronavirus pandemic, where an immense amount of patient data is being recorded that cannot feasibly be reviewed manually. A good structured and reliable system could be extremely useful for the prevention of the disease in obtaining data to avoid spreading, appropriate diagnosis, and diagnostic possibilities of proven benefit. Big data is also well known as a health management tool to prevent future risks, reduce unnecessary expenses, decrease health disparities, and encourage efficient use of material (antibiotics, beds, medications, etc.) [52]. However, without the data to generate it, its use becomes aspirational. For physicians, this tool can provide valuable information to guide options for certain patients, as shown in a study-oriented on the critical patient that observed that numerous systems can predict a wide variety of health conditions [26]. However, most of these studies were single-center studies, which limited the generalizability of results and conclusions.

The combination of RHIS and big data is especially useful for the analysis and evaluation of the health problems of, and policies for, specific groups, particularly the chronically ill and elderly. We must adapt the information technology or system to the specific needs of each group. For example, chronically ill young people might prefer wearables, whereas a combination of face-to-face care and virtual follow-up would work better for older patients. Not all technologies or information systems are equally effective for the management of health problems. Consequently, the training of health personnel accompanied by the development of appropriate programs may allow the data obtained from smart homes and wearable devices to be dedicated to casual or sporadic monitoring and be a valid source of data for establishing global strategies for specific groups.

The difficulty that older adults have in handling technology is widely known, often due to unclear instructions or poor support, and hence, their perceptions of technology must be recorded to maximize and facilitate its use in their daily activities [53]. Therefore, the data obtained from smart homes, especially those where the elderly reside, become vital to evaluate because projects can be created that allow them to better manage their problems, complications, and even comorbidities. However, a study [22] that focused on elderly individuals found limitations in the technologies and also found that their main use is monitoring healthcare and not as an intermediary for information.

### 4.3. Outcomes

While an array of partial evidence shows how certain HISs, PHRs, or clinical decision support (CDSS) technologies and systems have positive impacts on the effectiveness of health systems, joint (multiple information systems), representative, and longitudinal evidence from population samples is very scarce. Social research into the health, organizational, and healthcare policy effects of RHIS use should be considerably expanded. It is especially important to consider the relationships of complementarity between RHIS and the technologies of the second digital wave, such as big data or data-driven management, artificial intelligence (AI) and machine learning, and collaborative platforms, among others.

The implementation of information technologies and digital systems in healthcare tends to be rejected by the general public. This rejection is linked to the generalized idea that investment in these systems is made to the detriment of investment in people who provide face-to-face care to others (the classic model of health care). RHIS implementation is often top down, and this is rejected by professionals and patient associations, who perceive that technology is being prioritized over people. Consequently, the opinions of professionals on the timing of the RHIS implementation must be incorporated and complement the launch with the necessary information technology and IS support. Crucially, the neutrality of the technology can be affected by the implementation of a specific technique. RHIS implementation should occur while considering the maximization or minimization of any foreseeable positive or negative effects. On the other hand, we must work on the permeability and connection of health organizations regarding RHIS and its justification and explanation to society about its needs and benefits for health systems. As with other information technologies and systems, the effective implementation of an RHIS is not possible unless there is general acceptance by its potential users (healthcare professionals and the general public).

RHISs represent major cultural changes for healthcare professionals and the general public. RHISs are not developed in isolation. Rather, they are a more effective instrument for organizations and healthcare policy to promote citizen empowerment regarding their own health. Empowered by multiple practices of information generation and digital communication in healthcare, citizens seek to be cared for in accordance with new criteria governing the doctor–patient relationship, which no longer needs to be the traditional passive one. At the same time, many citizens, mainly—though not solely—older ones, will still seek traditional services. RHIS can be useful for segmenting these different needs into personal categories depending on the health status of individuals and for developing different care methodologies and policies.

RHISs also offer significantly innovative and disruptive alternatives for health system organizations. Within this context, complementarity between RHIS, big data, and AI is especially important for the development of digital health platforms. Digital spaces can provide infinite possibilities for agents to connect with one another, in which the traditional separation between the roles of professional and patient becomes blurred and the limitations of place, time, and connection between equals are largely overcome. Moreover, 20th-century hospitals and primary care centers may be partially replaced by 21st-century digital health platforms. These platforms would serve as digital intermediaries between healthcare or wellness providers (not necessarily healthcare professionals), and those seeking healthcare.

### 4.4. Efficiency of RHISs in the Prevention/Treatment of COVID-19 Transmission

The development of an RHIS intervention would also be useful during the COVID-19 pandemic. That is because the pandemic has generated a series of new data (data related to procedures, trips, the movement of people, immigration, etc.) on top of the data already existing in health systems. Thus, through digital surveillance evidence and unstructured data profiling, this new and large amount of raw data can be turned into useful big data. These data must also be represented in RHISs in order to make better decisions and to take advantage of other data generated by digital sources (e.g., social media, train routes, Google Trends, etc.). Thus, using the data obtained by RHIS, it would be possible, for example, to examine patterns of use in selected health services. This is the case of Singapore, where the data obtained by RHIS were used to predict the health service use levels and thus better understand the pattern and magnitude of the COVID-19 effect on the use of certain services [54]. Bangladesh used prepandemic RHIS to develop a model that would predict total health service utilization, including an estimate of health service use levels if the pandemic had not occurred [54]. This should provide very important data to assess costs and develop health policies based on the results, compared to those obtained by RHIS in the current pandemic. In China, a similar system was used to quantify the effect of the COVID-19 pandemic on the use of health services. In this case, detailed monthly data were used, which included data for previous years, for the year the pandemic started, and even for the periods after the various waves of the pandemic. These analyses show that RHIS data are of great significance for timely and effective tracking of the performance of the health system in low- and middle-income countries [55].

RHISs could be useful for COVID-19 surveillance. In Bulgaria, contact tracing has been implemented by RHIS. Therefore, when someone who has had close contact with a person with confirmed COVID-19, he or she is registered and has to be tested [56].

WHO has incorporated RHIS data standards into key projects such as immunization, HIV, malaria, tuberculosis, and reproductive, maternal, newborn, child and adolescent health (RMNCAH), and continues to include other data in its own digital health package to be able to report health data that has proven to be a key need [57], especially in the era of the COVID-19 pandemic. However, not everything is perfect in the use of RHIS in relation to COVID-19. For example, an RHIS may not be able to capture the full impact of the COVID-19 epidemic in populations that have health services that do not report data to it (nongovernmental or religious organizations), or in those in which health services are provided by the private sector. In addition, many countries (especially low- and middle-income ones) do not have systems in place for the routine assessment of data quality. These systems are often beset with data entry errors and with an inconsistent application of reporting definitions, due to a failure to use standards [58].

### 4.5. Policy Implications

In 2015, the WHO Regional Office for Europe developed a tool to guide the assessment of HISs and the development of a national health information strategy [59]. According to a survey of European members, it was agreed necessary and desirable to improve the integration of HISs at the national level. Better sharing of these health data allows for more and better comparative health research, international benchmarks, and national and EU-wide public health monitoring [60]. However, some countries lack the resources to implement the program properly or even specify the financial resources for the preparation of the program in the budget, which may challenge the desired integration. Participants in one study mentioned various other challenges that have different relevance to countries, such as data availability, opportunities for linking data sources, legal restrictions, technical restrictions, and institutional issues [59].

Several European nations are considered leaders in the use of electronic medical records (especially in primary care). In these, HISs have been used for much longer than in other nations of the world [61]. Nevertheless, RHISs continue to display a gap between recording, reporting, and the effective use of data; therefore, strengthening RHISs has become a global priority for tracking and addressing national health goals [62]. The operations of RHISs in low-income countries fall below the globally expected standard due to the production and use of poor quality data, or to not using high-quality data to make informed decisions [63,64]. Despite investment in RHISs in low- and middle-income countries, several problems still persist (technical, organizational, financial), thus preventing proper use of RHIS (incorrect data and nonuse of data already in the system) [65]. The use of RHISs in various low-income countries in Latin America and in Africa is associated with the most significant local public health problems, such as interventions to improve maternal and newborn health [66], or to reduce communication delays and improve quality of care via a tuberculosis laboratory information system in Peru [67]. RHIS data from the research and health policy community in Mozambique will help build sustainable long-term capabilities to manage and evaluate health conditions effectively [68]. In Ethiopia, the Ministry of Health and the Bill & Melinda Gates Foundation (Bill & Melinda Gates Foundation) launched “Operational Research and Coaching for Analysts” (ORCA) as a method for developing data collection and reporting [62].

Despite a large number of studies and reviews on HISs, contradictory results continue to be evidenced. This is because some parts of the systems are unpredictable, such as the users, the flow of information, and the settings [61]. Even if they present a number of problems, RHISs can help to strengthen policy decision making in local health systems, especially in low-income countries. Therefore, it is necessary to establish a suitable strategy based on the digitization of data processing, which allows indicator use to be simplified and reports to be saved and delivered, thus leading to a modern and effective data use structure.

To establish improvement strategies, it is necessary to know what the current problems and weaknesses in the evaluated studies are. Indeed, we can find various aspects in need of improvement within them, including the lack of a strategy for RHIS system implementation and evaluation [25,32]; the lack of financial, personnel, and equipment resources, making it impossible to correctly collect data capable of providing the best results [31,35,40]; data capture systems (software) that are not intuitive enough and require extra training, which hinders their use [22,25,35] and causes rejection by their users (especially doctors) [24,33,35]. Moreover, the lack of interoperability between systems (medical records, databases, etc.) further complicates the proper use of data [24,25,28,36]. Without adequate planning to overcome poor communication between technical, administrative, and health personnel [32], the results obtained from such data are only able to give an overview that is of little benefit to local entities [22,27,28,30,36].

By identifying such problems, strategies can be established to solve them. These strategies should be established and grouped by the specific determinants found: technical, organizational, and behavioral [63]. The reason for doing so is that it is practically impossible to generalize an answer within a single overall strategy.

Technical strategies: The records must be simplified in a standardized way to facilitate data entry. Ideally, creating intuitive software is an excellent choice that may even increase user acceptance. The development of tools to improve the results of poor RHIS data has been described in other studies with good results [65]. These tools could even allow data availability and usability to be improved (by both uploading new data and reviewing data that is already available), possibly by using cloud storage services to enable easy access from anywhere and by having a common standard that allows for interoperability of systems in different locations.

Organizational strategies: The management of resources is essential, of both those available and those needed, and that is why it is very important to have a protocol for project implementation before any project is actually carried out. It is necessary to assess what can be achieved with the financial and human resources that are presently available, as well as the possibility of making improvements by obtaining new resources. To solve organizational problems, projects must be correctly established from the start. This should include follow-up measures (based on variables) and evaluation so that any post-evaluation improvements can be made so as to enhance the use of the data obtained. In addition, the possibility of establishing a project monitoring and evaluation director position should be considered. This is because leadership within projects such as these is essential to guide, monitor, and resolve any issues that team members may have. These teams must be made up of professionals from the various areas participating in the project since this will help to create the right tools, which should be useful to all potential users, and also be easy to use.

Behavioral strategies: These aim to improve the staff members’ competence and motivation to collect, extract, and use data effectively [63]. One of the most commonly encountered problems is the participants’ (mostly health practitioners’) refusal to use an RHIS because they consider that using a new tool will not bring any benefits. This can be resolved through educational interventions to show the benefits of RHISs and by training staff to use them properly. These actions have been shown to improve staff members’ abilities to use data [65]. Here, a leadership figure is very important for the purpose of providing guidance during project rollout. This is because workshops and educational interventions do not always achieve the expected results, whereas the combination of leadership and motivation can have a powerful behavioral and organizational impact on data improvement [63].

In order to achieve the integration of issues, it is important to clearly understand what should be integrated, how it should be integrated, what activities should be considered, and the benefits that can be obtained. Through current technological advances, certain basic information system improvements can be demonstrated (providing quality data, data recordkeeping, legislative and technical infrastructure, and personnel improvements) that promote process integration in Europe. To accomplish this, proper leadership and good management are key to improving RHIS architecture and infrastructure [54].

### 4.6. Limitations

Although this systematic review was conducted according to the suggested methodology, we acknowledge that our study has some limitations. We searched four databases and focused only on systematic reviews, meta-analysis, and bibliometric analysis. Consequently, our search may not be exhaustive. On the other hand, the inferior quality scores based on AMSTAR-2 tools might reflect incomplete reports rather than unqualified review methods (see Table S4 on Supplementary Materials). Finally, the large number of publications required an optimized approach. However, we have ensured transparency by clearly outlining the process followed in the Methods Section. Therefore, we expect this review will only serve as a temporary system review and can be further updated as needed.

## 5. Conclusions

The use and development of plans for RHIS at the national level in European countries would also be desirable at the continental level. Our research is based on a variety of available related articles, showing the possibility of coordinating work in various areas and creating integrated recommendations.

Some strategies have been developed. However, some countries in the European region are still not working in concordance with the development of RHIS, including legislatively. To alleviate this obstacle, new global and international strategies should be planned, and the development of tools and mechanisms should be promoted in order to highly integrate platforms among European countries.

## Figures and Tables

**Table 1:** Data collection tools + data flow systems.

Ref.	Technical	Organizational	Data Needs, Data Production, Data Availability, Data Use	OUTPUT Good Quality of Information
Eden et al., 2016 [25]	(-) The changing nature of HIE across users, information systems, and organization contexts. (-) Lack of standard classification and description of HIE architectures. (-) Lack of a theoretical framework underpinning HIE implementation and evaluation.		(+) Strong privacy and safety policies and patient training; registered or online informed consent; approaches to identify patients.	
Medic et al., 2019 [26]	(+) Easy-to-use, (Human–computer interaction) HCI-centric interfaces during deployment		(+) Show justification for decisions and underlying data to clinical users	(+) Access and compilation of information at the patient level through retrospective studies via EHR
Gentil et al., 2017 [28]	(-) The influence of the technological infrastructure (HCE software for data extraction) on the scope of data collection projects. (-) The challenges of provider choice and initial purchase negotiations for EHR software.	(-) The nature of the data analyzed (coded or free text data) or privacy management is a major deterrent for GPs.	(-) Most of AP’s data collection projects were not limited to a specific geographic location within a country.	
		(+) The set of facilities, services, and products offered by networks that extract data sets from the data warehouse in the areas of medical research and public healthcare		
Reeder et al., 2013 [32]	(-) Future HSH and HCH research should explore how to capture and implement standardized measures reported by participants in PHR. (-) Family members should be included as participants.		(+) The incorporation of data on the activity of the elderly in clinical information systems for (1) preventive health self-management and self-monitoring, (2) IT strategies to connect multiple stakeholders.	(-) Lack of evidence that technology provides feedback to older adults for decision making in their daily activities or maintaining their own health
Anker et al., 2011 [21]				(+) Frequency of use, information, channels, and contents of the search; credibility of the information source, satisfaction with the information obtained
Marschollek et al., 2007 [22]			(-) Accessibility remains largely hypothetical for groups of older people, the most disadvantaged	(-) The quality and semantic accessibility of website content is a major issue.
Meidiawati et al., 2020 [40]	(-) Mobile applications should be associated with information that comes from the medical record, having a storage option so the data is more concise and can be viewed quickly.			
Alexander et al., 2020 [35]			(-) Administrative data are inherently limited due to the lack of clinical specificity for laboratory conditions and results. (-) New clinical data needs to be included in the EMRs.	(+) The roadmap has five areas and content areas that LTC leaders should use to make strategic and comprehensive approach decisions.
Mahmoudi et al., 2020 [30]			(-) Granular data elements should be implemented through text mining, merging them with smaller geographic units of analysis, or by encouraging health systems to collect these outstanding attributes.	(+) EMR encompasses a large repository of multidimensional data.
Mäenpää et al., 2009 [36]		(-) Lack of common rules and policies for sharing clinical data. Lack of a consistent strategic plan. This results in consequences at the level of the organization’s culture and resistance to change.		
Wisner et al., 2019 [27]	(+) Focus on best practices for physician input on IT design to ensure that the content of the preconfigured templates makes it clinically meaningful and organized in a way that supports clinical work	(+) Effectively integrate narrative notes into EHR as an organizational aspect of clinical practice	(+) Improves the knowledge of professionals through greater access and visibility to information, having it available to multiple users, with data integrity and readability, or automatic data entry.	(+) Collect and synthesize information through data sources to contextualize and synthesize the information for the general description of the patient and to support clinical work.

(+) implies acting as a driver; (-) implies acting as a barrier.

**Table 2:** New electronic data systems + motivation, training, and support.

Ref.	Technical	Behavioral	Organizational	OUTPUT Effective Use of Information for Decision-Making
Eden et al., 2016 [25]	(-) Contrasting evidence is lacking on barriers to the use of HIEs by function type or by architecture type. (-) Technology and user needs. (-) The optimal functionality of HIE is challenged by the lack of consistent classification and terminology of HIEs and by the changing nature of the sociotechnical systems involved.	(-) Information is lacking in the HIE to justify its use (perception of privacy and patient safety; incompatibility or population scope; competition with health systems; liability and negligence issues).	(+) The vision of information technology in health as sociotechnical systems characterized by dynamic interdependence and the co-evolution of technologies and the social contexts in which they are used. (+) Thoughtful implementation and workflow.	(+) Include end users in identifying key HIE functions.
			(-) Following organizational and workflow aspects.	
Gentil et al., 2017 [28]	(+) Offer to GPs simplified data extraction tools to minimize additional workload.	(+) Promote to GPs with financial benefits, training sessions (in data coding), feedback reports, and participation in research studies.	(-) The limited applicability and usefulness of EHR data for large-scale research purposes.	(-) By using a single software application, it limits interoperability issues and facilitates technical data analysis.
Reeder et al., 2013 [32]	(-) Market forces dictate access to technology and services. (-) Existing commercial lifestyle monitoring technologies may not be ready for large-scale deployment. (-) Information related to technology costs and sustainable reimbursement models is lacking.	(+) Involve and inform family members of older adults and stakeholders in the development of HSH or HCH technology.	(-) The communication gap between health sciences and technology researchers. This makes transferability difficult when trying to redesign business processes and change the organizational culture of organizations.	
Åkesson et al., 2007 [37]	(+) Cooperation between nursing professionals and software engineering is important in creating consumer applications.	(-) More research is needed to measure consumer digital experiences in health and the factors that influence them.		
Anker et al., 2011 [21]		(+) Keep in mind the following attitudinal aspects: locus of control, self-efficacy, desire, and intentions to have medical information, satisfaction with the doctor–patient relationship.		
Eslami Andargoli et al., 2017 [39]			(+) Overcome partial approaches and use more holistic approaches that consider content, process, and context.	
Marschollek et al., 2007 [22]	(-) Not much work is being performed on the design of the interface for the elderly or people with functional disabilities.	(-) Positive attitudes toward web-based communication on the part of older people have been contrasted. (-) Technological limitations.		
Meidiawati et al., 2020 [40]		(+) PHRs can encourage users to engage in healthy living behaviors		
Weir et al., 2012 [38]				(-) The recommendations emphasize clarifying the phenomenon of CPOE, avoiding reporting conclusions through subgroup analysis, developing theoretical models, including more quantitative evaluations of results.
Oluoch et al., 2012 [33]	(-) Technical infrastructure problems (electrical power, erratic Internet connectivity, and access to mobile phones) impede the implementation and effective use of EMR–CDSS.	(-) Limited computer skills of clinicians prevent effective use of EMR–CDSS.	(-) Failure to comply with reminders by providers prevents effective use of EMR–CDSS.	
Bayoumi et al., 2014 [23]			(-) Due to their wide range, they may be more subject to alert fatigue.	(-) More research is required to find out about the quality, relevance, and usability of decision support, and to study clinical outcomes and costs.
Alexander et al., 2020 [35]	(-) Lack of trust in HIT providers; lack of interoperability between systems; and lack of adaptation of IT to existing work patterns.		(-) Limited financial resources for LTC technologies; deficits in human capital to execute and maintain HIT; shortage of vital networks that support the adoption, use, and exchange of information through technology.	(-) Research is lacking in LTC activities under healthcare delivery systems.
Mahmoudi et al., 2020 [30]	(-) Machine learning methods vary substantially in their interpretation, creating barriers and impediments to clinical acceptance and their implementation in all health systems.			(+) The use of EMR data and machine learning methods has created a huge opportunity to refine risk prediction tools for readmission of risk groups.
Mäenpää et al., 2009 [36]	(-) Aspects related to usability, privacy, and confidentiality.	(+) Advances in computer skills, employee engagement, leadership, and organizational rules; formal and sustainable business model	(+) Political initiatives (a strategic framework, construction of an electronic health information infrastructure, and an implementation plan that takes the organizations into consideration).	
Ingebrigtsen et al., 2014 [31]		(+) Strong, visible, and proactive leadership of a clinical profile with technical IT skills in health and with previous experience in IT project management. (+) International educational initiatives to improve the scope and dissemination of IT competencies in health	(+) The positive impact of clinical leaders on successful IT adoption (cultivating necessary IT competencies, establishing mutual partnerships with IT professionals, and executing identifiable proactive IT behaviors).	
Baysari et al., 2016 [24]	(-) The usability of the system and the negative impact of these systems on workflow or efficiency		(-) Low acceptance of IT systems by individual, clinical, and organizational factors, including the setting between technology and the different ways physicians work	(+) IT interventions can be effective in improving the appropriate use of antimicrobials in hospitals.
Cresswell and Sheikh, 2013 [34]	(+) Technology has the potential to adapt (or be customized) to support changing needs and individual and organizational contexts of use.	(-) End-user resistance to the use of systems that are deemed inappropriate or that interferes with their values, aspirations, and roles.	(+) Research drawing on experience in disciplines or fields of knowledge that contribute to the study of technical, social, and organizational issues is essential to promote knowledge about organizational adoption and best practices for implementation.	
Wisner et al., 2019 [27]	(-) The structure of the EHR does not always match the way of thinking and working of nurses, generating additional work to integrate the use of the EHR into their complex and dynamic workflows.	(+) EHR improves some aspects of cognitive work.		(-) The EHR’s focus on data integrity, aggregation, and storage has produced large volumes of information that clinicians find difficult to navigate and synthesize, making clinically meaningful information less accessible and available.

(+) implies acting as a driver; (-) implies acting as a barrier.

**Table 3:** Use-related data for service improvements.

Ref.	Behavioral	Organizational	OUTCOMES RHIS Performance	OUTCOMES Health Systems Performance	IMPACT Health Status
Eden et al., 2016 [25]			(-) Some hospital systems are hesitant to share health data with competitors because they are worried about losing patients and their market share.		
Arditi et al., 2017 [29]				(+) Reminders can improve the quality of care in various settings and under various conditions	(-) There is no certainty that reminders improve patient outcomes as the evidence is minimal.
Medic et al., 2019 [26]	(+) Efficient and training just in time.	(+) Integrate CDS into clinical workflows without adding unnecessary additional work. (+) Evaluate the effectiveness and risks of CDS. (+) Provide ongoing feedback to clinicians. (+) Understand the ethical challenges for CDS. (+) Standardize the implementation.	(+) Machine-learning techniques depending on the selected problem and the types of data used.		
Gentil et al., 2017 [28]		(+) Involvement of government services, academic institutions, and software companies, financing long-term and wide-ranging data collection projects.	(+) The local network effect facilitates the diffusion of initiatives.		(+) AI can provide clinical decision support systems, providing capabilities to analyze free-text information through new natural language processing algorithms.
			(-) Using different software applications hampers data collection and adds interoperability issues.		
Åkesson et al., 2007 [37]				(+) ICT can improve the nurse-patient relationship and increase the welfare of patients. (+) ICT resources made consumers feel more confident and empowered, increased their knowledge, and improved their health status.	
Anker et al., 2011 [21]				(-) Future research should analyze how the search for health information influences health management.	
Eslami Andargoli et al., 2017 [39]			(+) Map existing health information systems and assess their integrity based on their response to what (content), how and when (process), and who and why (context).	(+) Overcome partial approaches and address more holistic approaches that consider the content, process, and context approach.	
Marschollek et al., 2007 [22]			(-) Health information systems continue to be used primarily in health care for monitoring purposes, not as information brokering.		
Meidiawati et al., 2020 [40]					(+) PHRs can be tools to monitor physical exercise, eating behaviors, and weight control to evaluate whether hypertension has been controlled based on measures and related laboratory results.
Weir et al., 2012 [38]				(+) CPOE is associated with improvements in medication errors.	
Oluoch et al., 2012 [33]				(+) With EMP–CDSS, a reduction in data errors, missed appointments, missed CD4 results, and patient waiting times was observed.	
				(-) With EMP–CDSS, a significant increase was observed in the time dedicated by physicians to direct patient care.	
Bayoumi et al., 2014 [23]			(-) Multidrug alert systems rarely target only those drugs known to have the greatest potential for clinical benefit or harm, decreasing the likelihood of clinical benefit.	(+) Process results (changes in laboratory control behavior or prescription).	(+) Clinical outcomes (adverse drug events and length of hospitalization). (+) Clinical results substitutes (hypoglycemia and blood sugar average time in therapeutic range for INRs).
Alexander et al., 2020 [35]		(+) Promoting policy drivers, implementing HIT benchmarking, and decision support in senior healthcare.	(-) Slow adoption of many of the clinical support HIT technologies by LTC facilities, developments around LTC HIT.	(-) Absence of longitudinal care plans for the elderly with care needs; lack of codesign of technology and related systems for the provision of care.	
Mahmoudi et al., 2020 [30]			(+) The use of big data and sophisticated machine learning methods improve the predictability of readmission risk models based on EMR data.	(-) Explainable machine learning methods need to be developed and implemented to establish clinical utility and inspire potential changes in practice patterns.	(-) Health systems are not yet systematically collecting data about social and environmental factors, readmission risk, or other adverse health events.
Mäenpää et al., 2009 [36]		(-) Evaluate the value of the services arising from the exchange of health information for various stakeholder groups, such as providers, key players, and employers.	(-) There is a lack of experiences and data on factors for the successful formation and sustainability of clinical data exchanges; development and implementation of a framework for a health information network.	(+) RHIS provides patient information in real time; improves communication and coordination within a region, and case management and consultation with colleagues; allows patient-centered care processes to be redesigned; enables empowerment and multidisciplinary teamwork.	(+) RHIS enables improved clinical efficacy through the access and sharing of clinical data, leading to better health outcomes.
Ingebrigtsen et al., 2014 [31]			(-) National “top-down” policies, legislation, and financing.		
Baysari et al., 2016 [24]			(-) The lack of comparative analyses of different IT interventions to assess their relative performance in improving prescribing. (-) The variety of study designs and outcome measures used to evaluate IT interventions prevented meaningful comparisons between different types of IT systems.	(+) IT interventions may be effective in improving the appropriate use of antimicrobials in hospitals.	(-) Variable evidence of the impact of IT interventions on health outcomes, such as mortality and length of stay.
Cresswell and Sheikh, 2013 [34]	(+) Early and ongoing user engagement, technology’s relative advantage and early demonstrable benefits, communication, close adjustment to organizational priorities and processes, training and support, effective leadership and change management, and partnership and financial considerations.	(+) The potential of numerous disciplines or bodies of knowledge on the study of technical, social, and organizational issues to facilitate the implementation and adoption of innovations in complex health service systems. (+) The dimensions “implementation and use/design of technology” are interrelated. Factors must adapt to compensate for the change.		(+) Technical, social, and organizational considerations are essential to ensure that technological innovations are useful and usable (care provision) and support organizations or systems (organizational functioning).	
Wisner et al., 2019 [27]		(+) Effectively evaluating the impact of EHRs requires the interactions between physician, technology, the environment, and the social system to be considered. (-) Workflows in the clinical setting and the use of EHR in real life are rarely linear and predictable.			

(+) implies acting as a driver; (-) implies acting as a barrier.

## Data Availability

No additional data available.

## Supplementary Materials

The following are available online at https://www.mdpi.com/article/10.3390/ijerph18094622/s1. Table S1. Summary of included studies’ characteristics. Table S2. Summary of included studies’ results. Table S3. Attribute of different success factors. Table S4. Quality assessment judgment using the AMSTAR 2 tool.

## Abbreviations

AI	Artificial Intelligence
AIDS	Acquired Immunodeficiency Syndrome
AMSTAR	A Measurement Tool to Assess Systematic Reviews
CAAS	Computerized Antimicrobial Approval Systems
CDSS	Clinical Decision Support System
CIS	Clinical Information System
CPOE	Computerized Provider Order Entry
DOI	Theory of Diffusion of Innovations
D-RHIS	Disease-specific RHIS
DSO	Data Scientists Office
EHII	European Health Information Initiative
EHR	Electronic Health Records
EMR	Electronic Medical Records
GP	General Practitioner
HCH	Home-based Consumer Health
HCI	Human–computer Interaction
HIE	Health Information Exchange
HIS	Health Information System
HIT	Health Information Technology
HIV	Human Immunodeficiency Virus
HSH	Health Smart Homes
ICT	Information and Communications Technology
INR	International Normalized Ratio (prothrombin time)
I-RHIS	Integrated RHIS
IT	Information Technology
LTC	Long-Term Care
PHR	Personal Health records
PRISM	Performance of Routine Information System Management
RHIO	Regional Health Information Organizations
RHIS	Routine Health Information System, Regional Health Information System
SS	Surveillance Systems
TOE	Theory of Organization and Environment
TPB	Theory of Planned Behaviour
UTAUT	Unified Theory of Acceptance and Use of Technology
WHO	World Health Organization
