# Supplementary material for: Routine Health Information Systems in the European Context: A Systematic Review of Systematic Reviews
Source: Int J Environ Res Public Health. 2021 Apr 27;18(9):4622. doi: 10.3390/ijerph18094622 (PMC8123776; doi:10.3390/ijerph18094622)
Supplement: Supplementary file 1 [file ijerph-18-04622-s001.zip › ijerph-1149899-supplementary.pdf]

Table S1. Summary of included studies' characteristics

Ref	Journal	Country/ Universe	System/Technology	Setting/Scenario	Databases	No of Studies	Study type	Meta-analysis/ Systematic review / Bibliometric analysis
Eden KB, Totten AM, Kassakian SZ, Gorman PN, McDonagh MS, Devine B, Pappas M, Daeges M, Woods S, Hersh WR. Barriers and facilitators to exchanging health information: a systematic review.	Int J Med Inform. 2016 Apr;88:44-51. doi: 10.1016/j.ijmedinf.2016.01.004. Epub 2016 Jan 24. PMID: 26878761; PMCID: PMC4778080	International (USA, Finland, Austria and Denmark)	Systems for health information exchange (HIE) between non-affiliated organisations	Emergency services, outpatient clinics and hospitals	Ovid MEDLINE, PsycInfo , CINAHL, and Cochrane Library databases from January 1990 to February 2015	19 studies	Interviews, focus groups, and observations	Systematic review of 9 multi-centre case studies, 11 cross-sectional studies, and 2 before-and-after studies
Arditi C, Rège-Walther M, Durieux P, Burnand B. Computer-generated reminders delivered on paper to healthcare professionals: effects on professional practice and healthcare outcomes.	Cochrane Database Syst Rev. 2017 Jul 6;7(7):CD001175. doi: 10.1002/14651858.CD001175.pub4. PMID: 28681432; PMCID: PMC6483307.	International (USA, Canada, France, Israel and Kenya)	Automatic reminders generated by a computer system and delivered on paper to improve compliance with acute and chronic disease management guidelines	All medical care settings, including hospitals and outpatient care	Cochrane Central Register of Controlled Trials, Cochrane Database of Systematic Reviews, Health Technology Assessment Database, Database of Abstract of Reviews of Effectiveness, National Health Service Economic Evaluations Database (NHS EED) in the Cochrane Library, MEDLINE,	35 qualitative and 34 quantitative synthesis studies	Individual and group randomised and non-randomised trials evaluating the impact of computer-generated reminders delivered on paper to healthcare professionals (one-component intervention) and of additional co-interventions (multi-component intervention) compared to habitual care or co-interventions without the	Cochrane review

					Embase, CINAHL, and Inspec up to 21 September 2016		reminder component	
Medic G, Kosaner Kließ M, Atallah L et al. Evidence-based Clinical Decision Support Systems for the prediction and detection of three disease states in critical care: A systematic literature review [version 2; peer review: 2 approved].	F1000Research 2019, 8:1728 (https://doi.org/10.12688/f1000research.20498.2)	International (USA, Canada, UK, Germany and France)	Clinical decision support systems (CDSS)	Critical care, including intensive and emergency care	PubMed, MEDLINE (PubMed), ClinicalTrials.gov, and Cochrane Database of Systematic Reviews from 1 January 2013 to 8 November 2018	20 shock (haemodynamic instability), 22 respiratory insufficiency, and 31 sepsis	Evidence-based studies, methods and outcome measures, which were used to determine the clinical effectiveness of CDSS in detection and prediction among three populations. Retrospective and prospective case series, prospective cases and controls.	Systematic review focusing more on diagnostic performance than on other results
Gentil, M., Cuggia, M., Fiquet, L. et al. Factors influencing the development of primary care data collection projects from electronic health records: a systematic review of the literature.	BMC Med Inform Decis Mak 17, 139 (2017). https://doi.org/10.1186/s12911-017-0538-x	International (Canada, USA, Netherlands, UK, Spain, Belgium, Italy, Australia, France, Malta, Sweden and Switzerland)	Clinical information system (CIS) used to provide care or support its delivery, such as the electronic health record (EHR)	Primary care	PubMed database from December 2015 to November 2016	140 articles and 28 websites	Qualitative study, using a standardised form, on the project and its stakeholders, the aim of which was to compare projects	Systematic review of studies analysing the project for EHR data collection from different primary care physicians
Reeder B, Meyer E, Lazar A, Chaudhuri S, Thompson HJ, Demiris G. Framing the evidence for health smart homes and home-based consumer health technologies as a public health intervention for independent aging: a systematic review.	Int J Med Inform. 2013 Jul;82(7):565-79. doi: 10.1016/j.ijmedinf.2013.03.007. Epub 2013 Apr 30. PMID: 23639263; PMCID: PMC3740158.	International (Austria, Canada, Finland, Italy, Luxembourg, Spain, Netherlands, Korea, UK, France, Japan and USA)	Health smart homes (HSH) and home-based consumer health (HCH) technologies to support ageing at home	Independent ageing	MEDLINE, CINAHL, SciVerse ScienceDirect, ACM Digital Library, and IEEE Xplore databases from January 1980 to October 2011	31 publications	Randomised controlled trial, non-randomised controlled trial, historical controlled trial	Systematic review of studies focusing on supporting independence (e.g., medication reminder systems) or preventing health events that threaten independence (e.g., activity-level

								monitoring systems)
Kerstin M. Åkesson, Britt-Inger Saveman & Gunilla Nilsson. Health care consumers' experiences of information communication technology—A summary of literature	International Journal of Medical Informatics 76 (2007) 633–645	International (USA, Canada, UK, Ireland, Sweden and Finland).	Electronic information and communication resources with reference to health and illness in the nurse-patient relationship	Digital interaction between the healthcare professional (nurse) and the citizen in the healthcare sphere	CINAHL, MEDLINE, and Cochrane, as well as a manual search from 1995 to 2004	12/113	Pilot tests and qualitative, descriptive and population sample studies	Systematic review to capture the subjective consumer experience of using health-related electronic resources and the absence of illness
Ashley E. Anker, Amber Marie Reinhart & Thomas Hugh Feeley. Health information seeking: A review of measures and methods	Patient Education and Counseling 82 (2011) 346-354	International (more than 5 countries)	Using the Internet and social media to seek health information, generally in parallel to information provided by a healthcare professional	Seeking and processing health information, basically about general health status	PsycInfo from 1978 to 2010	129/648	Cross-sectional studies/interviews; retrospective review or cohort studies; observations or interventions; experiments or quasi-experiments; longitudinal analysis	Systematic review to evaluate citizens' active health information seeking methods and measures
Amirhossein Eslami Andargolia, Helana Scheepers, Diana Rajendran & Amrik Sohal. Health information systems evaluation frameworks: A systematic review	International Journal of Medical Informatics (2017) 97: 195–209	International (more than 5 countries)	Contextual systems ("who" and "why") for health information system (HIS) evaluation	HIS evaluation	Scopus, ScienceDirect, PubMed, CINAHL+, and Web of Science from 1980 to 2015	20/582	Social relation, behavioural research, life cycle development systems, and mixed-method studies	Systematic review of HIS evaluation contexts
Michael Marschollek, Stefan Mix, Klaus-H. Wolf, Beate Effertz, Reinhold Haux & Elisabeth Steinhagen-Thiessen. ICT-based health information services for elderly people: Past experiences, current trends, and future strategies	Medical Informatics and the Internet in Medicine (2007) 32(4): 251-261	International (Germany and the European Union)	Digital health information services for the older population	Digital HIS aimed at the older population, and ageing-related health problems	PubMed from 2003 to 2007	20	Case studies, web analysis, and quantitative and qualitative studies	Literature review (20 articles plus European projects) and web analysis

Yosi Meidiawati, Kemal N. Siregar & Berly Nisa Srimayarti. Qualitative, quantitative and case Personal Health Records in Managing Hypertension: A Systematic Review	Indian Journal of Public Health Research & Development (2020) 11(7): 1495-1500	International (USA, Canada and France)	Personal health records (PHR) using mobile applications	Use of PHR in the management of hypertension-related healthcare practices and health risks	PubMed, JSTOR, EBSCOhost, ProQuest, and Google Scholar from 2009 to 2019	4/103	Quantitative studies and randomised clinical trials (RCT)	Systematic literature review using the PRISMA protocol
Charlene R. Weir, Nancy Staggersb & Tamara Laukerta. Reviewing the impact of computerized provider order entry on clinical outcomes: The quality of systematic reviews	Internacional Journal of Medical Informatics (2012), 81: 219-231	International (more than 5 countries)	Computerised provider order entries (CPOE)	Analysis of the effects of CPOE on healthcare organisation and health status outcomes	PubMed, CINAHL, Scopus, Cochrane, Inspec, and PsycInfo from 1987 to 2010	13/185	Economic, quantitative, qualitative, and case studies	Systematic literature review of quality
Tom Oluoch, Xenophon Santasb, Daniel Kwaroc, Martin Were, Paul Biondichd, Christopher Baileye, Ameen Abu-Hannaf & Nicolette de Keizerf. The effect of electronic medical record-based clinical decision support on HIV care in resource-constrained settings: A systematic review	International Journal of Medical Informatics (2012) 81: e83-e92	International (Kenya, Rwanda, Uganda, Botswana and Haiti)	Electronic medical records (EMR) for clinical decision support (CDS)	Use of EMR for CDSS in the management (quality and effectiveness) of HIV care in resource-constrained settings	MEDLINE, Embase, CINAHL, and Global Health Library up to January 2012	12/2095	Quantitative and qualitative studies on the use of EMR in CDSS, and their link to HIV patients' clinical data	Systematic literature review
Imaan Bayoumi, Mosab Al Balas, Steven M Handler, Lisa Dolovich, Brian Hutchison, Anne Holbrook. The effectiveness of computerized drug-lab alerts: a systematic review and meta-analysis	International Journal of Medical Informatics, 2014 Jun;83(6):406-15. doi: 10.1016/j.ijmedinf.2014.03.003.	International (not specified)	Computerised alerts about prescriptions for adult patients linked to physicians via EMR	All medical care settings, including hospitals, outpatient care, and older people's homes	Computerised Clinical Decision Support System Systematic Review (CCDSSR) and Medications Management through Health Information Technology	36 studies	RCT of computerised drug-lab alert systems	Meta-analysis and systematic review, with a search strategy combining search terms for medication management with computer- and technology-related terms, limited to intervention

					(MMIT) databases, which had searched MEDLINE, Embase, CINAHL, Cochrane Database of Systematic Reviews, and International Pharmaceutical Abstracts from 1974 to 27 March 2013			studies with a comparison group
Alexander GL, Georgiou A, Doughty K, Hornblow A, Livingstone A, Dougherty M, Jacobs S, Fisk MJ. Advancing health information technology roadmaps in long term care.	International Journal of Medical Informatics 2020 Apr;136:10408. doi: 10.1016/j.ijmedinf.2020.104088. Epub 2020 Jan 24. PMID: 32120318; PMCID: PMC7340178.	International (Australia, New Zealand, UK and USA)	Health information technology (HIT) for clinical support (laboratory, pharmacy, and radiology), which facilitates the collection and analysis of data relating to long-term care (LTC)	Care of older people, in the home and in institutional settings	Ovid MEDLINE from 2000 to 2018	99 studies	Exploration of 4 HIT roadmaps in LTC developed by members of the 4 countries involved, and development of an international LTC HIT roadmap.	Extensive systematic review with a limited number of search terms to support and underpin the assumptions made in the collaborative roadmaps
Mahmoudi Elham, Kamdar Neil, Kim Noa, Gonzales Gabriella, Singh Karandeep, Waljee Akbar K et al. Use of electronic medical records in development and validation of risk prediction models of hospital readmission: systematic review	BMJ 2020; 369 :m958	International (not specified)	CIS used to provide care or support its delivery, such as EMR	Hospital setting	Ovid MEDLINE, Ovid Embase, CINAHL, Web of Science, and Scopus from January 2015 to 1 January 2019	41 articles	Studies that developed and validated a predictive model for hospital readmission within 28 or 30 days from initial discharge	Systematic review of variation in the prediction of readmission for patients vs specific patient populations by examining the properties of the candidate characteristics based on EMR, the differences in

								performance between machine learning and traditional regression models, and in the quality of the studies
Mäenpää T, Suominen T, Asikainen P, Maass M, Rostila I. The outcomes of regional healthcare information systems in health care: a review of the research literature.	International Journal of Medical Informatics 2009 Nov;78(11):757-71. doi: 10.1016/j.ijmedinf.2009.07.001. Epub 2009 Aug 4. PMID: 19656719	International (UK, Finland, Austria, Canada, Greece, Denmark, France, Germany, South Africa and USA).	Regional HIS (RHIS), regional health information organisations (RHIO), disease specific RHIS (D-RHIS) and integrated RHIS (I-RHIS)	Hospitals, nursing facilities, clinics, private medical practices, pharmacies, laboratories, radiology centres, health departments, and possibly patients themselves	MEDLINE from 1966 to May 2008, CINAHL from 1982 to May 2008) Cochrane Library, and PubMed/MEDLINE from 2000 to December 2008	24 articles	Empirical research studies, mainly on types of regional health information system or network, from a functional perspective	Systematic review of HIT and the implementation of HIE
Ingebrigtsen T, Georgiou A, Clay-Williams R, Magrabi F, Hordern A, Prgomet M, Li J, Westbrook J, Braithwaite J. The impact of clinical leadership on health information technology adoption: systematic review.	International Journal of Medical Informatics 2014 Jun;83(6):393-405. doi: 10.1016/j.ijmedinf.2014.02.005. Epub 2014 Mar 3. PMID: 24656180	International (USA, Western Europe, Canada, Asia, South America, Australia and New Zealand)	CIS used to provide care or support its delivery, such as EHR	Hospitals, primary care, secondary care, and integrated care in all organisations	MEDLINE, Embase, CINAHL, and Business Source Premier from 1 January 2000 to 21 May 2013	32 studies	Qualitative studies, combined with quantitative results in some cases	Systematic review of empirical studies on the impact of clinical leadership on the results of HIT adoption by organisations providing patients with medical care
Baysari MT, Lehnbom EC, Li L, Hargreaves A, Day RO, Westbrook JI. The effectiveness of information technology to improve antimicrobial prescribing in hospitals: A systematic review and meta-analysis.	International Journal of Medical Informatics 2016 Aug;92:15-34. doi: 10.1016/j.ijmedinf.2016.04.008. Epub 2016 Apr 30. PMID: 27318068.	International (not specified)	Information technology (IT) to support adequate antimicrobial prescribing (independent CCDSS, CDSS integrated with the EMR of a hospital or CPOE, computerised antimicrobial approval	Hospitals	MEDLINE from 1950 to March 2015, Embase from 1947 to March 2015, and PubMed from 1966 to March 2015	40 articles	Before-and-after test designs, and the inclusion of a control group. Random allocation of providers, patients or units (e.g., rooms) to an IT intervention. Objective outcome measures (e.g.,	Systematic review of IT interventions for antimicrobial prescribing in hospitals, and meta-analysis to determine the impact of IT interventions on three outcome measures:

			systems (cAAS), and surveillance systems (SS)				length of hospital stay) or subjective measures with blinded assessment	appropriate use of antimicrobials, patient mortality, and length of hospital stay
Cresswell K, Sheikh A. Organizational issues in the implementation and adoption of health information technology innovations: an interpretative review.	Int J Med Inform. 2013 May;82(5):e73-86. doi: 10.1016/j.ijmedinf.2012.10.007. Epub 2012 Nov 9. PMID: 23146626.		eHealth applications for computerised decision support, electronic prescription, EMR	Organisational settings	MEDLINE, Embase, Cochrane Database of Systematic Reviews, Database of Abstracts of Reviews of Effects, Cochrane Central Register of Controlled Trials, Cochrane Methodology Register, Health Technology Assessment Database, Google, LILACS, IndMED, PakMediNet, National Research Register, ClinicalTrials.gov, Current Controlled Trials, and NHS EED from 1997 to 2010	13 systematic reviews	Clinical results, evidence relating to ways of promoting the effective development, implementation and routine use of eHealth applications in medical care settings	Interpretative review of a systematic literature review examining the effectiveness of eHealth applications to improve the quality and safety of healthcare, and organisational issues relating to HIT innovations
Wisner K, Lyndon A, Chesla CA. The electronic health record's impact on	Int J Nurs Stud. 2019 Jun;94:74-84. doi: 10.1016/j.ijnurstu	USA, Scandinavia, Australia,	EHR	Intensive care units for patients admitted to community or	MEDLINE/PubMed, CINAHL, and Embase; psychology –	18 articles	The Mixed Methods Appraisal Tool (MMAT) was used to appraise	Integrative review with a summary of experimental, non-

nurses' cognitive work: An integrative review.	.2019.03.003. Epub 2019 Mar 14. PMID: 30939418.	Austria and Canada		tertiary hospitals, hospitalisation units (paediatrics, neonatal intensive care, perinatal, medical surgery, oncology, orthopaedics, and critical care settings), practice settings, and the US Veterans Administration. The focus of the studies included US perceptions	Web of Science and PsycInfo; and information science – PubMed, CINAHL, Embase, Web of Science, and PsycInfo from 2004 to 2016		the quality of eligible studies	experimental, and theoretical data

Table S2. Summary of included studies' results

Ref	Data type and collection	Barriers	Facilitators	Results	Limitations	Implications: challenges and opportunities	Evaluation of information systems
Eden KB, Totten AM, Kassakian SZ, Gorman PN, McDonagh MS, Devine B, Pappas M, Daeges M, Woods S, Hersh WR. Barriers and facilitators to exchanging health information: a systematic review.	Patients' clinical and contextual information	Lack of information in HIE to justify its use (patients' perceptions of privacy and security (USA); population incompatibility or reach; competency in health systems; responsibility and negligence).	Detailed information about patients (robust policies on privacy and security; patient training; informed consent recorded online; approaches to identify patients, e.g., algorithms); reflexive implementation and workflow (user sign in;	The barriers and facilitators were grouped into information integrity, organisation and workflow, technology, and users' needs. Those sites with proxy users (e.g., nurses, registrars) in the workflow made greater use of the HIE.	There was insufficient evidence to compare the barriers to HIE use by type of function (query-based vs exchange via EHR) or by type of architecture (centralised or not).	Changing nature of HIE across users, information systems and organisation contexts.<br><br>Lack of a standard description and classification of HIE architectures.<br><br>Lack of a theoretical framework underpinning HIE implementation and evaluation.	Evidence of barriers to and facilitators of HIE use centres on end-users' perceptions of their experiences but does not include formal studies of usability.<br><br>Potential for biased samples, due either to deliberative sampling of stakeholders for interviews, or to low survey response rates.

Organisation and workflow (disruptive, non-integrated user sign in requiring too many clicks; proxy users; lack of technical support; cultural issues relating to information exchange; data entry in both EHR and HIE).<br><br>Technology and users' needs (too much/illegible information; lack of patient context; lack of data standards; the HIE is in competition with the existing hospital portal, which contains much more detailed information).	sociotechnical approach; continued training for providers and proxy users; continuous user feedback gathering; monitoring of access metrics and provider contribution; managing expectations in relation to the new HIE; interface and workflow designed for providers and proxy users; leadership; technical support).<br><br>Including end-users in the identification of key HIE functions (obtaining data directly from EHR vs query-based exchange), access to short reports or an alert before the full report or for when it becomes available on the HIE; sharing contextual notes, automatic integration with	HIT is seen as a set of sociotechnical systems characterised by the dynamic interdependence and co-evolution of technologies and of the contexts within which they are used. Further research is required to better understand this relationship.<br><br>Some hospital systems are hesitant to exchange health data with competitors because they are concerned about losing patients and market share.	Understanding of the optimal functionality of HIE is challenged by the lack of consistent HIE classification and terminology, and the changing nature of the sociotechnical systems involved.

			existing provider systems; inclusion of providers and proxy users in the interface design).				
Arditi C, Rège-Walther M, Durieux P, Burnand B. Computer-generated reminders delivered on paper to healthcare professionals: effects on professional practice and healthcare outcomes	Information was generally obtained from EMR or a computerised database			Reminders probably slightly improve quality of care.<br><br>Reminders alone (one-component intervention) probably improve quality of care compared to habitual care.<br><br>Adding reminders to one or more co-interventions (multi-component intervention) probably slightly improves quality of care compared to co-interventions without the reminder component.	The aim of reminders was to improve compliance with preventive guidelines (e.g., cancer detection tests, vaccination) and with acute and chronic disease management guidelines (e.g., annual follow-ups, laboratory tests, medication adjustment, advice).<br><br>It is uncertain whether reminders improve patient outcomes because there is little evidence.<br><br>None of the studies reported harm or adverse effects.	Reminders can probably improve quality of care in various settings under various conditions.	Quality of care as a result of changing care practice and the criterion for assessing quality of care (e.g., requesting a test or starting a treatment). In turn, improving patient outcomes, according to the evidence.
Medic G, Kosaner Kließ M, Atallah L et al. Evidence-based Clinical Decision Support	EHR (detection of health conditions, prediction of deterioration or		Retrospective studies and EHR use facilitate access to and the	CDS has evolved to encompass a multitude of techniques in	Most of the prospective studies reviewed covered	Integrating CDS into clinical workflows without adding unnecessary extra work (providing the right	Retrospective studies using large samples. In the three areas investigated, the number

Systems for the prediction and detection of three disease states in critical care: A systematic literature review [version 2; peer review: 2 approved].	adverse events, therapy guidance, up-to-date information for clinicians about new recommendation s or modifications, providing guidance when predicting clinical trajectories for different patient profiles)		collection of large amounts of patient-level information.<br><br>Displaying the rationale for decisions as well as underlying data to clinical users, would lead to better adoption.	machine learning (algorithms based on rules and simple regression models), which are dependent on the problem selected and the data types used.<br><br>A diverse range of models applied in different hospital settings trained to predict a range of health conditions (sepsis and septic shock, venous thromboembolisms, acute kidney injury, and surgical site infections).	individual centres. Very few were multi-centre ones.<br><br>Diversity in the identified CDSS makes it challenging to draw conclusions on methodology. Lack of comparisons between different classifiers within studies, especially for the indication of shock, adds to this challenge.	person with the right information in the right intervention format at the right point in his/her workflow through the right channel).<br><br>Developing tools and concrete proof-points able to assess the efficacy of CDS in the clinic. Providing clinicians with continuous feedback.<br><br>Easy-to-use interfaces centred on human-computer interaction (HCI) during implementation.<br><br>Efficient training that is available when needed.<br><br>Not overloading clinicians with CDS alerts (bundling alerts according to underlying conditions).<br><br>Understanding ethical challenges for CDS and risk assessment in every site before deployment.<br><br>Standardising implementation across organisations.	of retrospective studies exceed by far the number of prospective studies conducted in a clinical setting.
Gentil, M., Cuggia, M., Fiquet, L. et al. Factors influencing the development of primary care data collection	Primary care data from patients' EHR and unprocessed health records	The availability of many different software applications appears to	Primary care physicians' investment is often promoted with financial	The technological infrastructure (EHR software for data extraction) can influence the	No factors that might limit primary care physicians' participation	The choice of EHR vendor and the negotiations for the initial EHR software purchase were considered as the first major	Technological infrastructure, primary care physicians' roles, stakeholders in the data collection network of the

projects from electronic health records: a systematic review of the literature.		hinder data collection.<br><br>The use of different software applications increases the complexity of data collection and adds interoperability issues.<br><br>The nature of the data analysed (coded or free-text data) and the management of privacy are major deterrents for primary care physicians.	benefits, training sessions (data coding), feedback reports, and involvement in research studies.<br><br>Offering primary care physicians simplified data extraction tools to minimise the additional workload: regular feedback and reports on EHR data recording to better understand and manage their activity, their patient population, and their research.<br><br>The local network effect can facilitate the spreading of initiatives.	outreach of data collection projects.<br><br>Most primary care data collection projects were implemented nationally and were not limited to a specific geographical location within a country. However, some networks were based on location similarity.<br><br>Networks that extract datasets from the data warehouse facilitate data exploitation for researchers and offer a range of products and services in the areas of medical research and public healthcare.	were identified, e.g., privacy issues, lack of training and information.<br><br>The technical architecture of the IT system for data reuse was poorly detailed in all articles.<br><br>Most projects were linked to a single software application, thus limiting interoperability issues and technically facilitating data analysis.	challenges. Companies producing EHR software were requested to implement the data extraction tools.<br><br>The applicability and utility of EHR data for large-scale research purposes remains limited. Big data require a deep knowledge of the content and form of the data. Artificial intelligence can provide CDSS and abilities to analyse free-text information through new natural language processing algorithms.<br><br>The concomitant involvement of governmental services, academic institutions, and software companies to finance long-term and wide-ranging data collection projects.	main primary care data collection projects, and their prevalence worldwide was compared.
Reeder B, Meyer E, Lazar A, Chaudhuri S, Thompson HJ, Demiris G. Framing the evidence for health smart homes and home-based consumer health technologies as a public health intervention for independent aging: a systematic review.	Activity patterns ("lifestyle monitoring") in the home, heart rate	Gap in communication between technology and health science researchers in the area of health smart homes (HSH) and home-based consumer health (HCH)		Emerging studies drew conclusions about technology's function and improvement. Promising studies on the acceptability of technology and the viability of measuring behaviour and outcomes. Effective studies	Lack of solid evidence in relation to support for ageing in the home due to the study designs, sample size and other factors. Limited quality of effective studies (first tier)	IT strategies to connect multiple stakeholders interested in older people's social support.<br><br>Future HSH/HCH research should explore how to capture and implement compatible and standardised measures informed by participants	IT research based on theoretical frameworks and models to provide justification of hypothetical relationships and a guide for their verification. Validated, reliable instruments that are also conceptually consistent with the measures of interest (functioning and

technologies. This lack of communication hinders transferability by trying to redesign business processes and changing the organisational culture of organisations.<br><br>The designs of the studies do not address real-life contexts and are limited to evidence.<br><br>Market forces impose access to technology and services.<br><br>Existing commercial lifestyle monitoring technologies may not be ready for large-scale implementation.<br><br>Lack of information on technology costs and sustainable		(first tier) measured real outcomes, such as changes in cognitive levels and time outside the home, in participant samples that were large enough to calculate statistically significant results.	due to participant drop-out rates, non-randomised comparison groups, and the use of a historical control. There was effective evidence (second tier) of HSH/HCH having been implemented successfully within contexts outside their original settings.<br><br>Few studies included family members as participants or showed evidence of technology supporting social interaction.<br><br>Lack of evidence of technology providing feedback to older adults for decision-making in their daily activities or for looking after their own health.	(psychosocial issues and health behaviour) in PHR.<br><br>Incorporating older people's activity into CIS for 1) preventive health self-management and self-control, 2) IT strategies to connect multiple stakeholders: PHR for family and older-adult use; CIS for clinical use; and community health registries (CHR) for use by public health service organisations and older people.<br><br>Need for a developmental approach that supports older adults' needs and preferences instead of a technology-driven approach.<br><br>Involving and informing the relatives of older adults as well as stakeholders in the development of HSH/HCH technologies.<br><br>Longitudinal studies to develop methods for predicting functional deterioration based on changes in activity patterns and for determining the effect and impact of promising technologies.	instrumental activities of daily life).<br><br>Specific and significant indicators are required for home-based telemonitoring systems.

		reimbursement models.					
Kerstin M. Åkesson, Britt-Inger Saveman & Gunilla Nilsson. Health care consumers' experiences of information communication technology—A summary of literature	Clinical and non-clinical data, surveys with non-clinical quantitative and qualitative data (Likert and open questions)		Three themes to encourage digital contact were identified: support and help, education and information, and efficient, secure telecommunications. The lack of face-to-face meetings or privacy did not appear to be a problem.	Information and communication technologies (ICT) can improve the nurse-patient relationship and augment wellbeing for consumers. Consumers felt more confident and empowered, their knowledge increased and their health status improved due to the ICT resources.	Further research is required to measure consumers' digital health experiences and the factors that influence it.	Cooperation between nursing and software engineering professionals is important in order to create applications enabling consumers to care for their health.	In the future, the position of consumers will become more powerful, and that will require nurses to have more and better information at their disposal to care for increasingly empowered patients. Similarly, technology has positive effects on specific groups, such as chronic patients, consumers in remote areas, and carers.
Ashley E. Anker, Amber Marie Reinhart & Thomas Hugh Feeley. Health information seeking: A review of measures and methods	Clinical and non-clinical data, surveys with non-clinical quantitative and qualitative data (Likert and open questions)	Frequency of use and information, search channels and content, credibility of information source, satisfaction with the information obtained.	Sociodemographic: Age, ethnicity, gender, educational level, health literacy.<br><br>Attitudinal: locus of control, self-efficacy, desire and intentions to have medical information, satisfaction with the doctor-patient relationship.	There are varying samples, measures and designs to identify those who do or do not seek medical information.	Future research should look into how health information seeking influences health management.	The social and relational functions of health information seeking should be analysed in greater depth. To that end, more advanced measures and methods should be used.	Health information seeking has a number of implications that have a direct impact on care practice and its efficiency and efficacy. These derived results are the desire to get a second opinion, discussing the information with one's doctor, patient satisfaction, self-diagnosis, and treatment implications.
Amirhossein Eslami Andargolia, Helana Scheepers, Diana Rajendran & Amrik Sohal. Health information systems evaluation frameworks: A systematic	Quantitative, qualitative, and mixed sociodemographic and attitudinal data			Users/patients, nurses and designers/managers are the stakeholders identified the most in evaluation. Effective design, efficiency and	Overcoming partial approaches and addressing more holistic ones that take into account content, process, and context.	Mapping existing HIS and evaluating their integrity depending on their response to the five main evaluation questions: "what" or content, "how" and "when" or process,	The need to shift from basic content-based ("what") models towards more holistic context-based ("who" and "why") models of HIS.

review				security are the main objectives pursued by evaluation.		and “who” and “why” or context.	
Michael Marschollek, Stefan Mix, Klaus-H. Wolf, Beate Effertz, Reinhold Haux & Elisabeth Steinhagen-Thiessen. ICT-based health information services for elderly people: Past experiences, current trends, and future strategies	Quantitative, qualitative, and mixed sociodemographic and attitudinal data	The quality and semantic accessibility of website content is a major problem. Little work has been done on the interface design for older people or those with functional disabilities.	Older people’s positive attitudes towards web-based communication have been contrasted, but there are also technological limitations in practice.	In healthcare, HIS continue to be used mainly for the purpose of monitoring rather than information intermediation.	Many publications focus on health information services for specific diseases and on their quality and semantic accessibility, yet few deal with presenting and customising health information for older and disabled people.	Very often the specific needs of this target group are not met, and therefore accessibility remains largely hypothetical.	A strategy with five key points is proposed for the design of sustainable health information services for older people. Specifically, the five elements for older people are content, form of presentation, integration, costs, and evaluation.
Yosi Meidiawati, Kemal N. Siregar & Berly Nisa Srimayarti. Potential Use of Personal Health Records in Managing Hypertension: A Systematic Review	Clinical and sociodemographic quantitative data		PHR can encourage users to engage in healthy living behaviours.	PHR can be tools to monitor physical exercise, eating behaviours, weight control, and the extent to which hypertension has been controlled based on measures such as blood pressure and related laboratory results.	Mobile applications can be associated with information that comes from outside the clinical record and have storage so data are more concise and can be displayed quickly.	Research should be continued to develop more PHR prototypes, and their use and efficiency should be tested to determine how they can be used to control hypertension.	Patient health reports should work automatically with data quality and the necessary information standards. In that way, PHR can be controlled and integrated with other evaluation systems, and especially with HIS.
Charlene R. Weir, Nancy Staggersb & Tamara Laukerta. Reviewing the impact of computerized provider order entry on clinical outcomes: The quality of systematic reviews	Qualitative, quantitative, and case studies with sociodemographic and attitudinal data			The recommendations stress the need to clarify the CPOE phenomenon by avoiding the reporting of conclusions across	The quality of original CPOE study research was variable. Previous systematic reviews did not address the	CPOE is associated with improvements in medication errors.	CPOE should not be treated is a unitary concept, and reviews should try to perform more thorough sub-group analyses. Issues relating to the quality, functionality, and

				sub-group analyses and developing theoretical models, including more quantitative assessments of outcomes.	central issue of CPOE efficacy.		implementation of research should be more integrated into the analyses.
Tom Oluoch, Xenophon Santasb, Daniel Kwaroc, Martin Were, Paul Biondichd, Christopher Baileye, Ameen Abu-Hannaf & Nicolette de Keizerf. The effect of electronic medical record-based clinical decision support on HIV care in resource-constrained settings: A systematic review	Quantitative and qualitative studies with clinical, sociodemographic and attitudinal data	Infrastructure is a clear barrier to implementation. Infrastructural obstacles include unstable electrical power, loss of Internet connectivity, and access to mobile phones. Humidity, dust and security concerns are also relevant.		With EMR-CDSS use, there were found to be reductions in data errors, in missed appointments, in missed CD4 results, and in patient waiting time. However, some studies reported a significant increase in the time clinicians spent on direct patient care.	None of the papers described a strong (randomised controlled) design.	Technical infrastructure problems such as unreliable electrical power and erratic Internet connectivity, clinicians' limited computer skills, and failure by providers to comply with the reminders are key impediments to the implementation and effective use of EMR-CDSS.	Low literacy and poor training of health workers on the use of digital information systems is the biggest challenge.
Imaan Bayoumi, Mosab Al Balas, Steven M Handler, Lisa Dolovich, Brian Hutchison, Anne Holbrook. The effectiveness of computerized drug-lab alerts: a systematic review and meta-analysis	Alerts for prescribing a single drug (single drug systems) and several drugs (multi-drug systems)	Multi-drug alert systems are rarely for drugs known to have greater potential for clinical benefit or harm alone, which dilutes the probability of clinical benefit. Moreover, due to their broad scope, they may be more subject to alert fatigue.		Three types of outcome: 1) Clinical outcomes (adverse drug events and length of hospital stay); 2) Clinical surrogate outcomes (hypoglycaemia, mean blood sugar level, and time within the INR therapeutic range); and 3) Process outcomes (changes in laboratory control behaviour or in prescribing).	CDSS must show clinical outcome benefits, especially given the high cost of implementing and maintaining computer systems. Further research is required to make progress on the quality, relevance and usability of decision support, and to look into the clinical	The most immediate impact of computerised alerts would be on changes in process, especially when more prolonged monitoring is required to show the changes in clinical assessment criteria.	There was no evidence to support that drug-lab monitoring alerts improve clinical outcomes.

					outcomes and costs, since the evidence does not warrant a broadening of decision support in this sphere.		
Alexander GL, Georgiou A, Doughty K, Hornblow A, Livingstone A, Dougherty M, Jacobs S, Fisk MJ. Advancing health information technology roadmaps in long term care.	Essential clinical data available in EMR (vital signs, laboratory results, or surgical procedure complexity)	Limited financial resources for implementing and sustaining long-term care (LTC) technologies; a deficit of human capital to support the knowledge, skills, and experiences required to execute and maintain HIT; shortage of vital affiliations to support the adoption, use, and sharing of patient information through technology.<br><br>Lack of trust in HIT providers; lack of interoperability between systems; lack of IT adaptation to	Promotion of policy drivers, implementation of comparative evaluations of HIT and decision support in the medical care of older people (European Federation of Medical Informatics, Health Information Management Systems Society of Asia Pacific, International Medical Informatics Association).	Despite the slow adoption of many HIT for clinical support (laboratory, pharmacy, and radiology) by LTC facilities, LTC HIT-related developments may increase.<br><br>There are five domains and content areas for the roadmaps, which LTC leaders should use to take decisions on strategic and comprehensive approaches as they harness the potential of LTC HIT to address future challenges and opportunities.	There is a lack of research into LTC activities within medical care provision systems.<br>Absence of longitudinal care plans for older people with care needs; lack of co-design of technologies and systems related to care provision.	There are concerns about the challenges (health sector and social care) of caring for a growing number of older people. Technological innovations should provide higher levels of care and allow more and more older people to play an active role in their own care.<br><br>New clinical data need to be included in EMR (e.g., Braden Score, Comorbidity Score, Laboratory-Based Acute Physiology Score, and functional or frailty status on discharge).	There are no recommended guidelines for the evaluation of external clinical support systems. Further research is required to guide LTC HIT development in these domains and technological content areas, which LTC HIT experts emphasise in their roadmaps.

		existing work patterns.					
Mahmoudi Elham, Kamdar Neil, Kim Noa, Gonzales Gabriella, Singh Karandeep, Waljee Akbar K et al. Use of electronic medical records in development and validation of risk prediction models of hospital readmission: systematic review	Clinical, demographic, healthcare encounter history, functional status, and socioeconomic status data available from EMR, and key social and environmental data from unstructured data such as physicians' notes, using natural language processing to extract those data	Health systems are still not systematically collecting social and environmental factors (carer availability or housing instability) or the risk of readmission or other adverse health events.<br><br>Administrative data are inherently limited due to the lack of clinical specificity for conditions and laboratory results.<br><br>The interpretation of machine learning methods varies substantially, thus creating barriers and impediments for clinical acceptance and for their implementation	EMR encompass a large repository of multi-dimensional data. Machine learning methods are capable of using the exhaustive set of data elements for consideration.	With the emergence of big data and sophisticated machine learning methods in healthcare, the number of predictive models of hospital readmission has improved the predictive ability of readmission risk models based on EMR data compared with that of other models using all other available datasets (administrative or survey data).	Most studies did not take into account salient socioeconomic features, failed to calibrate their models, and lacked careful assessment of the clinical utilities and implementation of the developed tools. The evolution of the field requires development and implementation of interpretable machine learning methods to establish clinical usefulness and inspire potential changes in practice patterns.	The use of EMR data and machine learning methods has created an enormous opportunity for further refinement of readmission risk prediction tools applicable to risk groups. The implementation of sufficiently granular data elements via text mining should be considered, merging them with smaller geographical units of analysis (census tract or neighbourhood level), or encouraging health systems to collect these salient attributes.<br><br>The contribution of sophisticated machine learning methods (neural networks) in medicine needs to be investigated.	The paucity of studies that provide information on the implementation and clinical utility of these models in the hospital setting leads to a substantial void in how these models can improve care coordination and discharge planning across readmission risk strata.

		across all health systems.					
Mäenpää T, Suominen T, Asikainen P, Maass M, Rostila I. The outcomes of regional healthcare information systems in health care: a review of the research literature.	Clinical data from the healthcare sphere, school clinical data, outpatient visit data, hospital discharge data, emergency department visit data, and laboratory data	Little experience of or data on the factors that contribute to the successful formation and sustainability of exchanges; the development and implementation of a framework for a health information network.<br><br>Non-existence of common rules and policies to share clinical data; non-existence of a consistent strategic plan.<br><br>Aspects relating to usability, privacy, and confidentiality.<br><br>Organisational culture and resistance to change.	Advancement of computer skills, employee commitment, leadership, formal organisational rules, and a sustainable business model.<br><br>Policy initiatives (developing a strategic framework, building an electronic health information infrastructure, and an implementation plan that takes organisations into account).	RHIS allow clinical effectiveness to be improved through clinical data access and exchange; they provide appropriate patient information in real time; they improve communication and coordination within a region; they allow the patient-centred care process to be redesigned; they improve case management and consultation with colleagues; they enable empowerment and multidisciplinary teamwork to better understand the patient situation and context, which leads to better health outcomes.	The different phases of RHIS development. The reported methodologies appear to be heterogeneous, which limits their comparability.	Integration is a way of developing HIS and new organisational models of collaboration that meet the needs of the population.<br><br>The most difficult challenge is that related to assessing the value of services that emerge from HIE to various stakeholder groups such as providers, players, and employers.	Different combinations of methodologies were used and the sample sizes were either small or not mentioned. The most common types of study design were survey research and case studies. Triangulation and qualitative methods can make a valuable contribution to the further improvement of evaluation research in medical informatics.
Ingebrigtsen T, Georgiou A, Clay-Williams R, Magrabi F, Hordern A, Prgomet M, Li J,	Data corresponding to performance and clinical measures	"Top-down" national policies, legislation and funding.	Strong, visible, and proactive leadership, of a clinical profile	Leaders' explicit IT knowledge.	Low or very low quality of the studies. While there are	There is a positive impact of clinical leaders on successful IT adoption in healthcare organisations.	The outcome measures varied widely, with a direct or indirect (respondents'

Westbrook J, Braithwaite J. The impact of clinical leadership on health information technology adoption: systematic review.	(patient outcomes), and degrees of success from HIT adoption (e.g., performance or safety)		with technical HIT skills and prior experience of IT project management.	International educational initiatives to enhance the scope and spread of HIT competencies.	guidelines aimed at improving the reporting of evaluation studies in HIT, accepted standards for critical appraisal of disparate studies are not readily available.	Clinical leaders who aim for improvements in the processes and quality of care should cultivate the necessary IT competencies, establish mutual partnerships with IT professionals, and execute identifiable proactive IT behaviours to achieve expected outcomes.	perceptions) assessment of the◎ degree of successful adoption being most common.
Baysari MT, Lehnbom EC, Li L, Hargreaves A, Day RO, Westbrook JI. The effectiveness of information technology to improve antimicrobial prescribing in hospitals: A systematic review and meta-analysis.	Specific information about the patient or from the EMR, guidelines or information about antimicrobial resistance profiles, pathology/microbiology results, and antimicrobial orders	Little acceptance of IT systems due to individual, clinical, and organisational factors, including the fit between technology and physicians' different ways of working.<br><br>System usability or the negative impact of such systems on workflow or efficiency.		IT interventions can be effective for improving the appropriate use of antimicrobials in hospitals, although better quality studies are needed to confirm this conclusion.<br><br>Varying evidence of the impact of IT interventions on health outcomes, such as mortality and length of hospital stay.	The range of study designs and outcome measures used to evaluate the IT interventions prevented meaningful comparisons between different types of IT system from being made. There is little available evidence to assist organisations in making informed choices about IT solutions for antimicrobial prescribing.	The impact of interventions would have been greater if more prescribers had used them regularly.<br><br>The lack of comparative analysis of different IT interventions to evaluate their performance in relation to improved prescribing leaves hospitals and governments with limited information to guide their decisions on the choice of IT interventions.	Conducting in-depth interviews with prescribers to explore users' needs and preferences with regard to antimicrobial prescribing support, and incorporating the findings into future system design, would not only increase the probability of acceptance, but also ensure that systems address problematic areas and are easier to use and to integrate into current practice.
Cresswell K, Sheikh A. Organizational issues in the implementation and adoption of health information technology	Organisational issues in relation to HIT innovations	End-users resist the use of systems that are viewed as inadequate or as	Early and ongoing user involvement, relative advantage of the technology and early demonstrable	The numerous disciplines or bodies of knowledge that contribute to the study of technical, social, and	Much of the available evidence concerning organisational issues in relation	The complex relationship between different technical, social and organisational dimensions means that there is no prescriptive approach to	The retrieved studies were qualitatively synthesised, and data were extracted relating to: (1) specific care settings and contexts; (2) skills,

innovations: an interpretative review. https://www.sciencedirect.com/science/article/pii/S1386505612001992?via%3Dihub		interfering with their values.	benefits, a close fit with organisational priorities and processes, training and support, effective leadership and change management, co-involvement, and financial consideration seem to be particularly important.<br><br>The technology should have the potential to be adapted (or customised) to support changing needs and individual/organisational contexts of use.	organisational issues are rich in potential to facilitate implementation and adoption of innovations in increasingly complex health service systems.<br><br>Technical, social, and organisational considerations are essential in ensuring that technological innovations are useful and usable (care provision), and support the organisations or systems (organisational functioning).<br><br>These dimensions are inter-related (implementation and use / technology design). Other factors should be adapted to compensate for the change.	to HIT innovations is anecdotal and retrospective in nature, stemming from single organisational experiences of implementing a specific application. These tend to be descriptive accounts, without much attention to relevant theoretical considerations, which makes drawing generalisable lessons from such reports difficult.	"successful" implementation. The emergence of unintended consequences may mean that strategies need to be adapted on an ongoing basis. This may require careful balancing between organisational demands (resources), social demands (user requirements) and technical demands (interoperability and performance).<br><br>Research employing experience in disciplines or bodies of knowledge that contribute to the study of technical, social, and organisational issues is central to furthering knowledge on organisational adoption and generalisable best practices for implementation.	knowledge, experience, attitudes, and values of individuals (clinicians, healthcare managers, and patients); (3) the characteristics of tools (such as adaptiveness); and (4) environmental factors, tasks, goals and their inter-relationships.
Wisner K, Lyndon A, Chesla CA. The electronic health record's impact on nurses' cognitive work: An integrative review.	Clinical data from EHR	The EHR focus on data integrity, aggregation, and storage has produced large volumes of information that clinicians found	The process of collecting and synthesising information through data sources, and contextualising and synthesising	Five topics were identified to describe how nurses and other clinicians perceived and used the EHR. These were: 1) forming and maintaining an	The MMAT was used to appraise the quality of studies, which ranged between 1 and 3 (on a scale from 0 to 4). The quality of	Effective evaluation of the impact of EHR requires the interactions between the clinician, technology, the environment, and the social system to be taken into account. Workflows in the clinical environment	The instruments used in the quantitative and mixed-method studies included the NASA task load index (NASA-TLX), an HIS monitoring instrument, the Information Systems

	difficult to navigate and synthesise, thus making clinically meaningful information less accessible and available.<br><br>The EHR structure is not always aligned with how nurses think and work, which creates additional work when it comes to integrating the use of EMR in their complex and dynamic workflows.	information is considered central to the process of forming an overview of the general description of the patient and supporting clinical work.<br><br>Cognition was improved by greater visibility or access to information, having available information for multiple users, integrity, legibility or data legibility, or automating data entry.	overview of the patient, 2) cognitive work of navigating the EHR, 3) use of cognitive tools, 4) forming and maintaining common ground and a shared understanding of the patient, and 5) loss of information and professional domain knowledge.<br><br>The EHR improves some aspects of cognitive work.	9 studies was considered moderate (MMAT score ≥3), and that of a further 9 was low. One point was deducted from half of the qualitative studies because they did not address the researcher's positioning.	and the use of EHR in real life are rarely linear or predictable.<br><br>Using a framework of human factors and sociotechnical systems, future research should focus on understanding how nurses retrieve, organise, synthesise, and communicate information; how they achieve and maintain clinical understanding and awareness of the situation when they use the EHR; and exploring IT design to support cognitive work.<br><br>How to effectively integrate narrative notes into the EHR as an organisational aspect of clinical practice; evaluating transfer and monitoring tools to align them to how nurses think and work; and focusing on best practices for clinicians' contributions to IT design in order to ensure that preconfigured template content is clinically relevant and organised in a way that supports clinical work.	Expectations and Experiences (ISEE) survey, a technology acceptance model adapted from Masrom and an e-learning survey, and surveys developed by researchers. Interviews, focus groups, observations, think-aloud and retrospective think-aloud sessions, and artefact analysis were used alone or in combination with the mixed-method and qualitative studies.

Table S3. Attribute of different success factors.

System/Technology Quality Attributes	Information Quality Attributes	Usage System/Technology Attributes	User Satisfaction Attributes	Individual Impact Attributes	Organisational Impact Attributes
Decision support HIS [22,28,29,36,39,40].	Strengths and weaknesses [39].	Limited applicability for large scale [27,28].	Lack of knowledge [28,33].	increase of knowledge [37].	Reduction of medication errors/adverse events [23,38].
Upgradeable systems [22,28].	Clinical support [24,35].	Learning time consuming [33,35].	People's skills and organizations' abilities [24-27,37,39].	Perceived improvement [27,32].	Reduction on data errors [33].
Lack of strategic planning [25,30,34,36].	Incomplete information [26,38].		Generational preferences [22,32,35].	Management elderly [22,32,35].	Quality of care [24,27,29-31,33].
Lack of organizational culture [21,30,32,34,36].	Accessibility [27].		Empowerment [37].	Positive impact in IT & leadership [24,30,34].	Preventive guidelines [29].
Lack of tech infrastructure [24,25,28,31,33–35].	Impediment to contextualizing and synthesizing information [27].		Satisfaction with doctor-patient relation [21].	Cost-effective and timesaving [24,28,34,36,37,40].	Better prescriptions [24].
Lack of policies [36].			Better approaches [27,39].	IT adoption causes better clinical decisions [24,27,30,34].	Better performance [23,26,40].
Usability [21].			Resistance to change [24,31,34,36].	Encourage healthy behaviours [40].	Predictive model [26,31].
Computer-generated reminders [29,33].			Promote training sessions [28,34].		Better clinical results [23,36].
Privacy, security, and confidentiality [30].					Medical specialties participating [23,25,26,28–30].
Strategic decision development -organizational hierarchies [34,35].					Allows implementation/planning [26,34,36].
					Better communication / coordination [22,25,27,34,36,37].
					Reduction of waiting list [33].

Table S4. Quality assessment judgment using the AMSTAR 2 tool.

Author, year	AMSTAR score																AMSTAR score summary				Quality of the review
	1	2	3	4	5	6	7	8	9	10	11	12	13	14	15	16	N	C*	Y	NA	
Eden et al., 2016 [25]	n	n	n	n	y	n	y	n	y	n	na	na	n	n	na	y	9	3	4	3	Critically low
Arditi et al., 2017 [29]	y	y	y	y	y	y	y	y	y	y	y	n	y	y	n	y	2	1	14	0	Low
Medic et al., 2019 [26]	y	n	y	y	n	n	y	y	n	n	na	na	n	n	na	y	6	3	7	3	Critically low
Gentil et al., 2017 [28]	n	y	y	y	y	y	n	n	n	n	na	na	n	n	na	y	7	3	6	3	Critically low
Reeder et al., 2013 [32]	n	n	y	n	y	n	n	n	n	n	na	na	n	n	na	y	10	5	3	3	Critically low
Åkesson et al., 2007 [37]	n	n	n	y	n	n	n	y	y	n	na	na	n	n	na	y	9	3	4	3	Critically low
Anker et al., 2011 [21]	n	n	n	n	n	n	n	n	n	n	na	na	n	n	na	y	12	5	1	3	Critically low
Andargoli et al., 2017 [39]	n	n	n	y	n	n	n	n	n	n	na	na	n	n	na	y	11	4	2	3	Critically low
Marschollek et al. 2007 [22]	n	n	n	y	n	n	n	y	n	n	na	na	n	n	na	n	11	4	2	3	Critically low
Meidiawati et al., 2020 [40]	y	n	n	y	n	n	n	n	n	n	na	na	n	n	na	y	10	4	3	3	Critically low
Weir et al., 2012 [38]	n	y	n	y	y	y	y	y	n	y	na	na	y	y	na	y	3	1	10	3	Low
Oluoch et al., 2012 [33]	y	y	n	y	y	y	y	y	n	n	na	na	n	n	na	y	5	2	8	3	Critically low
Bayoumi et al., 2014 [23]	y	y	y	y	y	y	y	y	y	n	y	y	y	y	y	y	1	0	15	0	High

Alexander et al., 2020 [35]	y	n	n	n	n	n	n	n	n	n	na	na	n	y	na	y	10	5	3	3	Critically low
Mahmoudi et al., 2020 [30]	y	y	n	y	y	y	y	y	n	n	na	na	n	n	na	y	5	2	8	3	Critically low
Mäenpää et al., 2009 [36]	y	n	n	y	n	n	n	n	n	n	na	na	n	n	na	n	11	4	2	3	Critically low
Ingebrigtsen et al., 2014 [31]	y	y	n	y	y	y	n	y	n	y	na	na	n	y	na	y	4	3	9	3	Critically low
Baysari et al., 2016 [24]	y	y	n	y	y	y	y	y	n	n	y	y	y	y	y	y	3	1	13	0	Low
Cresswell and Sheikh, 2013 [34]	y	n	n	y	y	y	n	n	n	n	na	na	n	n	na	y	8	4	5	3	Critically low
Wisner et al., 2019 [27]	y	y	y	y	n	n	y	y	n	n	na	na	n	y	na	n	6	2	7	3	Critically low

C*: Critical weakness.
